# Characterization, in-vitro biological and antimicrobial testing of replacing Sr/Ca in wollastonite (Ca_1 − x_ Sr_x_ SiO_3_) glass-ceramics

**DOI:** 10.1038/s41598-026-36649-1

**Published:** 2026-02-11

**Authors:** H. K. Abd El-Hamid, Gehan T. El-Bassyouni, Amira M. M. Amin, Abeer A. Abd El-Aty, Emad M. M. Ewais, Esmat M. A. Hamzawy

**Affiliations:** 1 Refractories, Ceramics and Building Materials Department, National Research Centre (NRC), El-Buhouth St., Dokki, Cairo, 12622 Egypt; 2 Ceramics Materials Department, Refractories, Central Metallurgical Research and Development Institute (CMRDI), Helwan, Cairo, PC87 Egypt; 3 Chemistry of Natural and Microbial Products Department, National Research Centre (NRC), El-Buhouth St., Dokki, Cairo, 12622 Egypt; 4 Glass Research Department, National Research Centre (NRC), El-Buhouth St., Dokki, Cairo, 12622 Egypt

**Keywords:** Wollastonite, Strontium, Glass-ceramics, Cytotoxicity, Antibacterial, Antifungal, Biotechnology, Materials science, Microbiology

## Abstract

Strontium-doped wollastonite glass-ceramics with varying Sr concentrations (0.125, 0.25, and 0.50 wt%) were successfully synthesized via a melt-quenching technique and comprehensively evaluated for structural, physical, and biological performance, including in vitro biocompatibility, antimicrobial activity, and cytotoxicity. Characterization using DTA, XRD, and FE-SEM/EDX confirmed progressive hydroxyapatite (HA) formation following 28 days of immersion in simulated body fluid (SBF), with XRD revealing a reduction in wollastonite peaks and distinct HA signals, particularly in Sr-containing samples. FTIR spectra showed intensified phosphate absorption bands, while FE-SEM/EDX analyses highlighted a morphological shift from plate-like structures to needle-like HA crystals. Strontium doping was found to play a key role in guiding HA crystallization pathways while supporting wollastonite’s intrinsic isovalent cation exchange capacity. Among all compositions, the W3Sr sample (0.5 wt% Sr) exhibited the best balance of mechanical properties and biological efficacy. Biologically, the material demonstrated dose-dependent antifungal activity against *Aspergillus niger* (11–18 mm IZD) and *Fusarium solani* (10–16 mm IZD), no antibacterial activity, and excellent cytocompatibility with BJ1 human fibroblasts. Overall, this Sr-doped wollastonite system shows strong potential for next-generation orthopedic and dental applications, combining Sr-mediated bioactivity, mechanical strength, antifungal efficacy, and cell compatibility.

**Supplementary Information:**

The online version contains supplementary material available at 10.1038/s41598-026-36649-1.

## Introduction

Calcium phosphate (CP) and calcium silicate (CS) biomaterials are widely utilized in orthopedic applications due to their enhanced osteogenic potential and compatibility with bone regeneration processes^1^. Wollastonite (CaSiO₃), a calcium silicate ceramic, is recognized as a bioactive material for bone tissue engineering owing to its pronounced bioactivity and controlled degradation kinetics^2^. Its popularity in glass-ceramic systems stems from distinctive properties that differentiate it from other silicate compositions, including its ability to promote osteogenesis while exhibiting non-cytotoxic behavior. However, pure wollastonite faces limitations as a load-bearing bone substitute due to inherent sintering challenges, which compromise porous structural integrity and result in inadequate mechanical strength^3^. To address these limitations, strategic ionic substitutions (e.g., Sr^2+^, Zn^2+^, Cu^2+^, Mg^2+^) have been integrated into biomaterial formulations, aiming to enhance physicochemical characteristics and biological performance^4^. Studies demonstrate that introducing bioactive ions such as strontium, zinc, copper, and magnesium significantly enhances osteogenic capacity, positioning these modified biomaterials as promising candidates for advanced bone repair solutions^5^.

Recent studies on doped wollastonite materials highlight their enhanced functionality for biomedical applications, particularly in bone regeneration and antimicrobial activity. Copper-doped wollastonite exhibits a distinct antimicrobial hierarchy, most effective against gram-negative bacteria, followed by fungi, then gram-positive bacteria. Furthermore, copper doping (3–5%) significantly improves fracture healing, promoting complete bone restoration in rats^6,7^. Other dopants also show promise: hematite (Fe₂O₃) adds magnetic functionality for bone fillers and hyperthermia; ZnO substitution enhances degradability and bioactivity; MnO₂ doping (0.5 wt%) enables full bone mineralization; and gold nanoparticles contribute immunomodulatory effects that accelerate regeneration. Collectively, these advanced wollastonite composites, synthesized primarily via wet chemical methods, demonstrate significant potential as multifunctional biomaterials^8–14^.

The substitution of calcium with strontium in biomaterials such as orthopedic cements and bioactive glasses has attracted increasing attention. Both calcium and strontium are divalent cations with similar ionic radii (Ca^2+^: 1.00 Å; Sr^2+^: 1.18 Å) and naturally accumulate in bone tissue, allowing for easy interchange within glass systems. This chemical compatibility, combined with strontium’s beneficial effects on bone metabolism, makes it a promising candidate for applications in bone tissue engineering and regenerative medicine^8,9^. This substitution enhances glass degradation rates and accelerates hydroxyapatite formation, while facilitating the sustained release of Sr²⁺ ions. These ions have been demonstrated to stimulate osteoblast activity and inhibit bone resorption, synergistically improving bone regeneration efficacy^15^.

Strontium (Sr) has emerged as the predominant element for doping silicate bioceramics due to its dual role in enhancing bioactivity and compatibility. Sr-doped bioceramics consistently demonstrate bioactive and biocompatible behavior while retaining non-cytotoxic properties across varying silicate compositions^16,17^. Their therapeutic efficacy stems partly from ionic dissolution products that modulate osteogenic pathways. As a divalent cation, Sr is a naturally occurring trace element in human bone, where it promotes osteogenesis and suppresses bone resorption during remodeling. Mechanistically, Sr enhances osteoblast differentiation by upregulating bone-related gene expression and alkaline phosphatase (ALP) activity, while concurrently inhibiting osteoclast formation. The substitution of SrO for CaO in wollastonite (CaSiO₃) via wet precipitation methods induces notable changes in the material’s physical properties, broadening its applicability in bone tissue engineering and regenerative therapies^8,15^.

*Pelepenko et al.*^18^, demonstrated that substituting calcium (Ca^2+^) with strontium (Sr^2+^) induces structural expansion in glass networks due to Sr²⁺’s larger ionic radius (0.113 nm vs. Ca²⁺’s 0.099 nm), thereby altering the material’s in vitro behavior. This substitution accelerates glass degradation and hydroxyapatite formation while releasing Sr^2+^ ions, which are known to enhance bone regeneration. Similarly, *El Damrawi et al.*^19^, reported that SrO incorporation improves surface adhesion and mechanical hardness in Sr-doped glasses, attributed to Sr^2+^’s lower electronegativity compared to Ca²⁺, which strengthens interfacial bonding. *Abdelghany et al.*^20^, synthesized SrO-substituted borate glasses (replacing CaO and NaO) via thermal treatment and evaluated their bioactivity and bone-binding capacity. Their findings revealed enhanced solubility in Sr-doped glasses and glass-ceramics, attributed to the formation of Sr-phosphate phases, which exhibit greater solubility than their calcium-phosphate counterparts^20^. The researchers observed that corrosion rates and weight loss in these materials increased with SrO incorporation, driven by the higher solubility of Sr-apatite compared to Ca-apatite. Moreover, strontium’s relatively low electronegativity facilitates charge balance within the glass matrix, stabilizing Si–O–Sr bonds. This structural modification promotes rapid formation of well-defined calcium-phosphate (Ca–P) species, enabling efficient apatite layer development and greater substitution of Ca²⁺ ions in Sr-doped systems^21–23^.

The novelty of this work lies in the rational, application-driven design of a phase-pure Sr-substituted wollastonite ceramic that simultaneously integrates controlled Sr²⁺ release, tunable degradation behavior, enhanced in vitro bioactivity, and selective antifungal performance within a single material platform. Importantly, antifungal activity in Sr-substituted wollastonite is reported here for the first time and is systematically correlated with Sr substitution level, phase evolution, and ion-release kinetics, providing clear mechanistic insight. A carefully controlled solid-state synthesis route ensures high phase purity and uniform microstructure, enabling reliable structure–property–biological relationships while maintaining cytocompatibility. Overall, this study introduces a multifunctional ceramic that unifies strontium-driven bioactivity with intrinsic antifungal functionality, addressing a critical unmet challenge in implant-associated fungal infections and demonstrating strong potential for next-generation bone regeneration and tissue engineering applications. This work introduces a new direction in Sr-doped wollastonite biomaterials by integrating three essential features: (1) precise atomic-level structural engineering, (2) improved osteogenic potential, and (3) targeted antifungal activity. These attributes together form a pioneering platform with strong promise for advancing regenerative medicine. The material was thoroughly characterized through thermal analysis of precursor glasses, X-ray diffraction (XRD), and field emission scanning electron microscopy with energy-dispersive X-ray spectroscopy (FE-SEM/EDX) to assess the microstructure of the heat-treated glass-ceramics. In vitro evaluations included bioactivity testing via cell culture, cytocompatibility assessment through cytotoxicity assays, and antimicrobial testing against both bacterial and fungal strains.

## Materials and methods

### Materials synthesis

This study involved the partial substitution of calcium with strontium in stoichiometric wollastonite (Ca₁₋ₓSrₓSiO₃), with Sr/Ca ratios of Ca₀.₈₇₅Sr₀.₁₂₅, Ca₀.₇₅Sr₀.₂₅, and Ca₀.₅₀Sr₀.₅₀. The raw materials included: Limestone (Samalut, Egypt; composition: CaO: 55.7 wt%, SiO₂: 0.15 wt%, MgO: 0.10 wt%, Al₂O₃: 0.22 wt%, IL: 44.02 wt%), Silica sand (Gulf of Suez coast, Sinai, Egypt; composition: SiO₂: 99.4 wt%, Al₂O₃: 0.3 wt%, Fe₂O₃: 0.1 wt%), Strontium carbonate (SrCO₃, 99.9% purity, ≤ 55.0 ppm trace metals; Scientific Laboratory Supplies, UK), Sodium carbonate (Na₂CO₃, Fluka Analytical, Sigma-Aldrich, Germany), added to reduce melting temperatures. Precisely weighed batches were melted in a platinum crucible using a Vecstar electric furnace (UK) at 1400 °C for 2 h (W0) and 1440 °C for 2 h (W1Sr, W2Sr, W3Sr). The melts were periodically stirred to ensure homogeneity before being cast into preheated stainless steel molds. The resultant glasses were immediately transferred to a preheated annealing muffle furnace at 480 °C, held for 1 h, and then cooled to room temperature at a controlled rate of 30 °C/hour by switching off the furnace. Table 1 summarizes the oxide composition (in wt%), melting temperatures, and formula for each synthesized glass-ceramic batch.

**Table 1:** Formulation and oxide composition of synthesized glass-ceramic batches.

Sample	Constituents in oxide wt%					Formula	Product
	CaO	SrO	SiO_2_	*Na_2_O	Melting Temp. (℃)		
(W0)	48.28	----	51.28	10	1400	CaSiO_3_	Bulk glass
(W1Sr)	35.43	11.46	53.12	10	1440	Ca_0.875_Sr_0.125_SiO_3_	Bulk glass
(W2Sr)	32.85	20.23	46.92	10	1440	Ca_0.75_Sr_0.25_SiO_3_	Bulk glass
(W3Sr)	20.04	37.03	42.94	10	1440	Ca_0.50_Sr_0.50_SiO_3_	Bulk glass

*Added over 100 wt% oxide wt%.

### Materials characterization

#### Design and evaluation of heat treatment protocols via differential thermal analysis (DTA)

Powdered alumina acted as a reference standard for differential thermal analysis (DTA) of glass powder samples subjected to heat treatment at 1000 °C for 2 h. Thermal profiles were acquired using a Perkin-Elmer DTA-7 instrument. The resultant DTA thermograms were analyzed to identify the critical transformation temperature required for converting the parent glass into its glass-ceramic phase.

#### X-ray diffraction (XRD)

The crystalline phases of the sintered samples were characterized using a Bruker D8 Advance X-ray diffractometer (Germany) with Cu-K_α_ radiation of λ = 0.15418 nm. Scans were performed across a 2θ range of 5° to 70°, employing a step size of 0.02° and a dwell time of 1 s per step. Phase identification was carried out via a search-match algorithm using the International Center for Diffraction Data (ICDD, Newtown Square, PA, USA) database.

#### Fourier-transform infrared spectroscopy (FTIR)

Fourier-transform infrared (FTIR) spectroscopy was performed using a JASCO FT/IR-4600 spectrometer (USA) to characterize the functional groups in the synthesized materials. The analysis spanned a wavenumber range of 400–1600 cm⁻¹. For sample preparation, powdered specimens were blended with potassium bromide (KBr) in a 1:100 (sample: KBr) mass ratio and pressed under 5 tons/cm² pressure to generate uniform pellets. FTIR spectra were recorded at 20 °C with a spectral resolution of 2 cm⁻¹, enabling precise identification of molecular vibrational signatures.

#### Scanning electron microscopy (FE-SEM/EDX)

The bioactive potential and microstructural evolution of sintered composites were assessed using a field emission scanning electron microscope (FE-SEM; FEI Quanta 250 FEG, Netherlands) equipped with energy-dispersive X-ray spectroscopy (EDX). Operating at 20 kV, the system characterized surface morphology, elemental composition, and apatite mineralization. Prior to analysis, glass samples were crystallized via heat treatment at 1000 °C for 2 h, followed by soaking in simulated body fluid (SBF) at 37 °C for 28 days to estimate bioactivity. To optimize imaging, samples were gold-sputtered using an Edwards S150A coater (England) under 0.1 torr vacuum with 1.2 kV voltage and 50 mA current, enhancing surface conductivity. FE-SEM/EDX analysis revealed structural features, and apatite crystal formation, enabling a comprehensive study of post-SBF surface mineralization and microstructural changes.

#### High-resolution transmission electron microscope (HR-TEM)

The structural characteristics and morphological structure of the particles were analyzed using a high-resolution transmission electron microscope (HR-TEM; JEOL JEM-2100, Japan) operating at 200 kV. For imaging, an aqueous dispersion of the particles was drop-cast onto a carbon-coated copper grid, air-dried at ambient temperature, and subjected to microscopic analysis to evaluate morphology and lattice structure.

### In vitro biological evaluation

In the present study, the in vitro bioactivity evaluation in simulated body fluid (SBF) was performed under static conditions, in which each sample was immersed in a fixed volume of freshly prepared SBF. The SBF was prepared according to the protocol reported by *Kokubo and Takadama*^24^, in which the elemental and ionic concentrations are adjusted to closely match those of human blood plasma and the pH is maintained at 7.4. This protocol has been widely validated for assessing apatite-forming ability, and it considers the stability of ion concentrations and pH in as-prepared, conventional, and newly developed SBFs, as well as their behavior after storage at 37 °C ± 0.5 for various periods. The sample mass-to-SBF volume ratio was maintained at a constant ratio throughout the experiments. Following ISO/FDIS guidelines, discs were placed in sterile non-reactive polypropylene containers with SBF volume adjusted proportionally to the sample’s surface area (Sa). The sealed containers were incubated at 37 °C for predetermined intervals (1, 3, 7, 14, and 28 days). Post-immersion period, samples were extracted from the SBF, rinsed with deionized water to terminate residual reactions, and air-dried at ambient temperature prior to analysis via FE-SEM/EDX. This analysis aimed to assess the formation and distribution of calcium phosphate phases on the sample surfaces. The SBF immersion protocol is detailed in Eq. (1)^25^.

1 $$V_{{SBF}} = Sa/10~\left[ {mL} \right]$$

The sample surface area (Sa) used in Eq. (1) was calculated based on the geometrical dimensions of the specimens. For disc-shaped (cylindrical) samples, the total exposed surface area was determined using the following Eq. (2):

2 $${\mathrm{Sa}} = {\mathrm{2}}\pi {\mathrm{r}}^{{\mathrm{2}}} + {\mathrm{2}}\pi {\mathrm{rh}}$$

Where r is the radius of the disc and h is its thickness. This calculation includes both flat surfaces (top and bottom) as well as the lateral (side wall) surface, assuming that the entire sample was exposed to the SBF solution during immersion. The calculated surface area was then used to determine the required SBF volume according to Eq. (1).

#### Mechanical properties

The compressive strength of the composites was evaluated by testing three cylindrical specimens (12 mm diameter × 19 mm height) per formulation, fabricated using a uniaxial pressing machine (Paul-Otto Weber Maschinen- und Apparatebau, GmbH, Germany) under 25 kN/mm² pressure. Testing was performed with a LLOYD Instrument (Model LR 10 K) in compliance with ASTM C142-10 (Standard Test Method for Monotonic Compressive Strength of Advanced Ceramics at Ambient Temperature)^26^. Moreover, flexural strength measurements were performed on bar-shaped specimens (5 × 5 × 60 mm) using a universal testing machine (Tinius Olsen 25 ST, UK). A loading rate of 0.5 mm min⁻¹ was applied, with a support span of 40 mm, following the ASTM C1161–02 standard^27^. The flexural strength values were calculated using Eq. (3).

3 $$\:\sigma = \:\:\frac{3}{{2\:}}\frac{{FL}}{{bd^{2} }}\:\:$$

Where F: Maximum force (load).

L: Span width (40 mm).

b : Width of sample (mm).

d : Thickness (mm).

#### Bulk density and apparent porosity

The bulk density of the samples was measured at ambient temperature via Archimedes’ method, employing water as the immersion medium^28^.

#### Weight loss

To ensure uniformity, samples were prepared in standardized dimensions. Initial sample weights (M_1_​) were recorded using an analytical balance. Specimens were then immersed in simulated body fluid (SBF) and removed at predetermined intervals (1, 3, 7, 14, and 28 days) to track weight loss kinetics. Post-immersion, samples were gently rinsed with deionized water, air-dried to eliminate residual moisture, and reweighed (M_2_​). Percentage weight loss was calculated using Eq. (4). This protocol was repeated across all five immersion periods to evaluate temporal degradation trends^29^.

4 $${\text{Weight loss }}\left( \% \right){\text{ }} = \left( {{\mathrm{M}}_{{\mathrm{1}}} - {\mathrm{M}}_{{\mathrm{2}}} } \right)/{\mathrm{M}}_{{\mathrm{1}}} \times {\text{ 1}}00{\text{ }}\%$$

Where, M_1_ denotes the specimen’s initial mass pervious soaking, and M_2_ denotes its dry mass following the soaking period.

#### Variation of pH and ions degradation

Variations in pH were observed together with the amounts of P, Ca and Si ions released into the biological solution (SBF). The pH value was measured with an electrolyte-type pH meter. Inductively coupled plasma optical emission spectroscopy (ICP-OES; Agilent 5100VDV, Germany) was employed for the evaluation of Ca, P and Si ions, with a detection limit of 0.01 mg L^-1^^30,31^.

#### Surface characterization

The sample surfaces were characterized using FTIR, XRD, and FE-SEM/EDS to assess the formation of calcium phosphate layers following a 28-day immersion in SBF.

### Cytotoxicity test

The BJ1 human fibroblast cell line used in this work was sourced from the Bioassay–Cell Culture Laboratory, National Research Centre, Dokki, Cairo 12,622, Egypt. Cytotoxicity was assessed using the MTT assay, a standard and widely accepted method for determining cell viability, proliferation, and potential toxicity. In this assay, mitochondrial dehydrogenases in metabolically active cells reduce the yellow tetrazolium salt MTT (3-methylthiazole-2-yl-5-diphenyltetrazolium bromide) into insoluble dark blue formazan crystals, which reflects the number of viable cells.These insoluble crystals form selectively in viable cells due to their intact membranes and are later solubilized for quantification. All procedures were conducted under sterile conditions in a biosafety class II laminar flow cabinet (Baker, SG403INT, Sanford, ME, USA). Cells were maintained in DMEM-F12 culture medium enriched with 1% L-glutamine, 10,000 U/mL potassium penicillin, 10,000 µg/mL streptomycin sulfate, and 25 µg/mL amphotericin B, incubated at 37 °C in a 5% CO₂ atmosphere^32^. Following a 10-day batch culture, cells were transferred to 96-well micro titer plates at a density of 10,000 cells per well using complete growth medium. The plates were incubated for 24 h at 37 °C in a 5% CO₂ humidified water-jacketed incubator (Sheldon, TC2323, Cornelius, OR, USA)^32^. After removing the medium, fresh serum-free medium was introduced, and cells were either left untreated (negative control) or exposed to the test substance at final concentrations of 500, 250, 125, and 62.5 µg/mL. Post 48-hour incubation, 40 µL of MTT solution (2.5 µg/mL) was added to each well. The medium was then aspirated, and plates were incubated for an additional 4 h at 37 °C under 5% CO_2_. To solubilize the formazan crystals, 200 µL of 10% sodium dodecyl sulfate (SDS) in deionized water was added to each well, followed by overnight incubation at 37 °C. Doxorubicin, serving as a positive control, exhibited 100% lethality at 100 µg/mL under identical conditions. Statistical analysis was performed using SPSS 11 software: an independent t-test compared sample results to the negative control, while probit analysis determined IC₅₀ and IC₉₀ values. Cell viability percentages were calculated using Eq. (5)^33^.

5 $$\left[ {\left( {{\text{Reading of extract }}/{\text{ Reading of negative control}}} \right){\text{ }}{-}{\text{ 1}}} \right]{\text{ }} \times {\text{ 1}}00$$

### Antimicrobial studies

The antibacterial and antifungal efficacy of three glass and glass ceramic samples was evaluated against the base material using the agar diffusion method^34^. Tested pathogens included Gram-positive *Staphylococcus aureus* (ATCC 6538), Gram-negative *Escherichia coli* (ATCC 25922), and filamentous fungi (*Aspergillus niger* ATCC 18666, *Fusarium solani* NRC18). Microbial suspensions were prepared to standardized concentrations (1 × 10⁶ spores/mL for fungi; 1 × 10⁸ CFU/mL for bacteria). For testing, 1 mL of each suspension was inoculated into plates containing 50 mL of sterile nutrient agar (bacteria) or potato dextrose agar (fungi). Samples (100, 50, 25, and 12 mg) were placed in ~ 9 mm diameter wells on the agar surface and incubated at 4 °C for 120 min to facilitate compound diffusion. Bacterial plates were incubated at 37 °C for 24 h, while fungal plates were incubated at 28 °C for 72 h. All antimicrobial agar diffusion assays (including the stability test) were performed in triplicate (*n* = 3) for each sample concentration and microbial strain. Inhibition zones were measured at three distinct points, averaged, and reported as Mean ± standard deviation of three independent replicates using Microsoft Excel.

#### Stability test

Samples demonstrating antimicrobial activity were assessed for their stability against pathogens over 3–6 days of incubation^3^.

Ethical approval for the cytotoxicity and antibacterial activity study protocols was granted under reference number 10420125, which exempted the proposals from further ethical review.

### Statistical investigation

The data were obtained from independent experiments and are presented as mean ± standard deviation (SD) with *n* = 3. Statistical comparisons among groups were performed using a two-way ANOVA with Tukey’s post-hoc test, with *p* < 0.05 considered statistically significant. The standard deviation values are provided in each table, and the error bars represent the corresponding SD.

## Results and discussion

### Characterization of materials

**Fig. 1:**
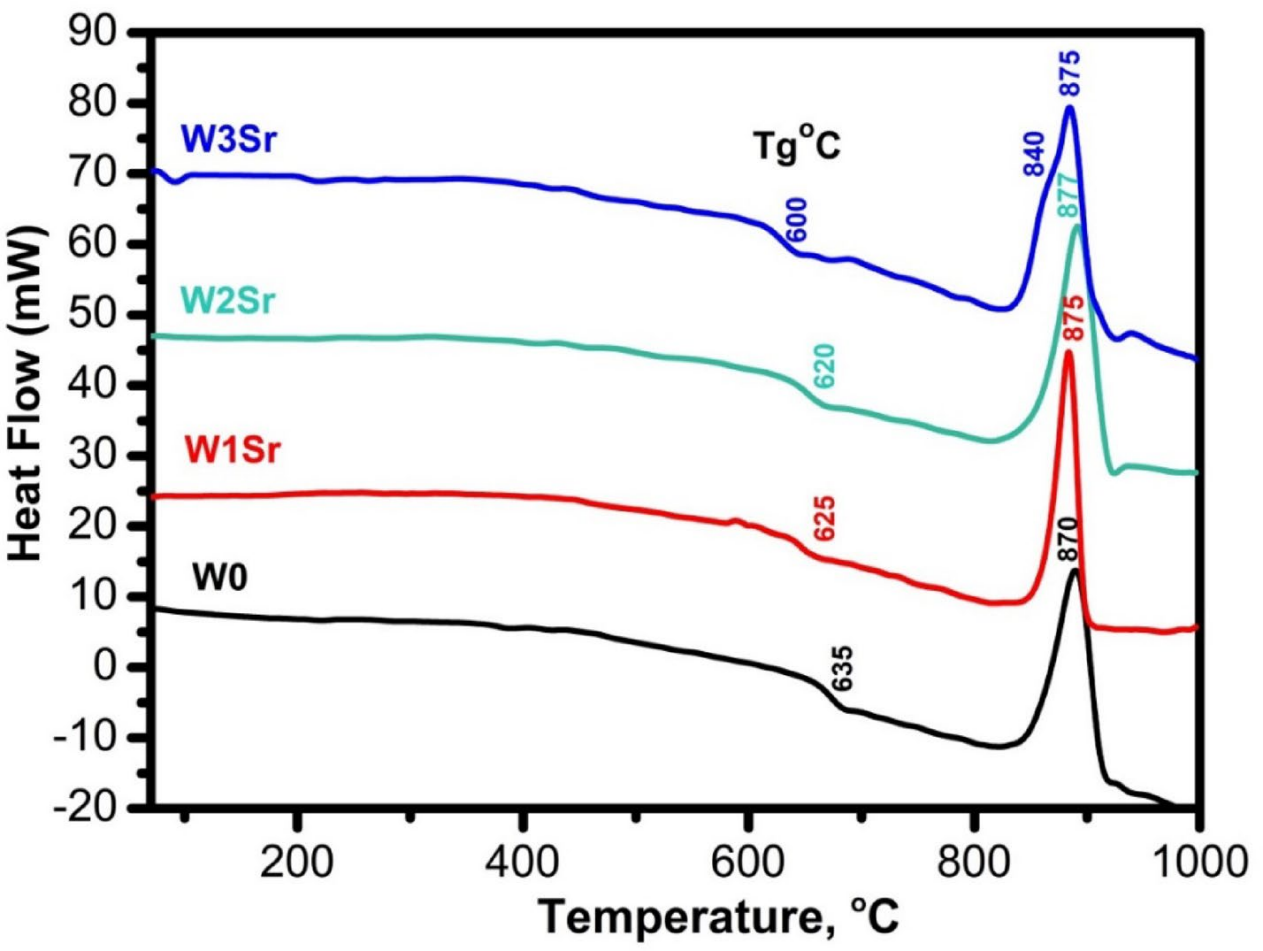
DTA curves of the glass samples.

Differential thermal analysis (DTA, Fig. 1) revealed the thermal properties of the glass samples. The glass transition temperature (Tg℃) slightly decreased from 635 °C to 600 °C with increasing SrO content which is attributed to the role of Sr^2+^ as a network modifier. By disrupting the bridging Si–O–Si bonds, Sr^2+^ increases the formation of non-bridging oxygens (NBOs), which weakens the overall network structure. This reduction in connectivity enhances chain mobility and lowers the thermal energy needed for the glass-to-liquid transition, resulting in a decreased Tg^35^. In contrast, the primary crystallization temperature (Tc℃) remained consistent between 870 °C and 877 °C for all compositions. However, a distinct shoulder at 840 °C on the curve for the W3Sr sample indicates the formation of a secondary, strontium-rich crystalline phase, revealing increased complexity in its crystallization behavior^11^.

**Fig. 2:**
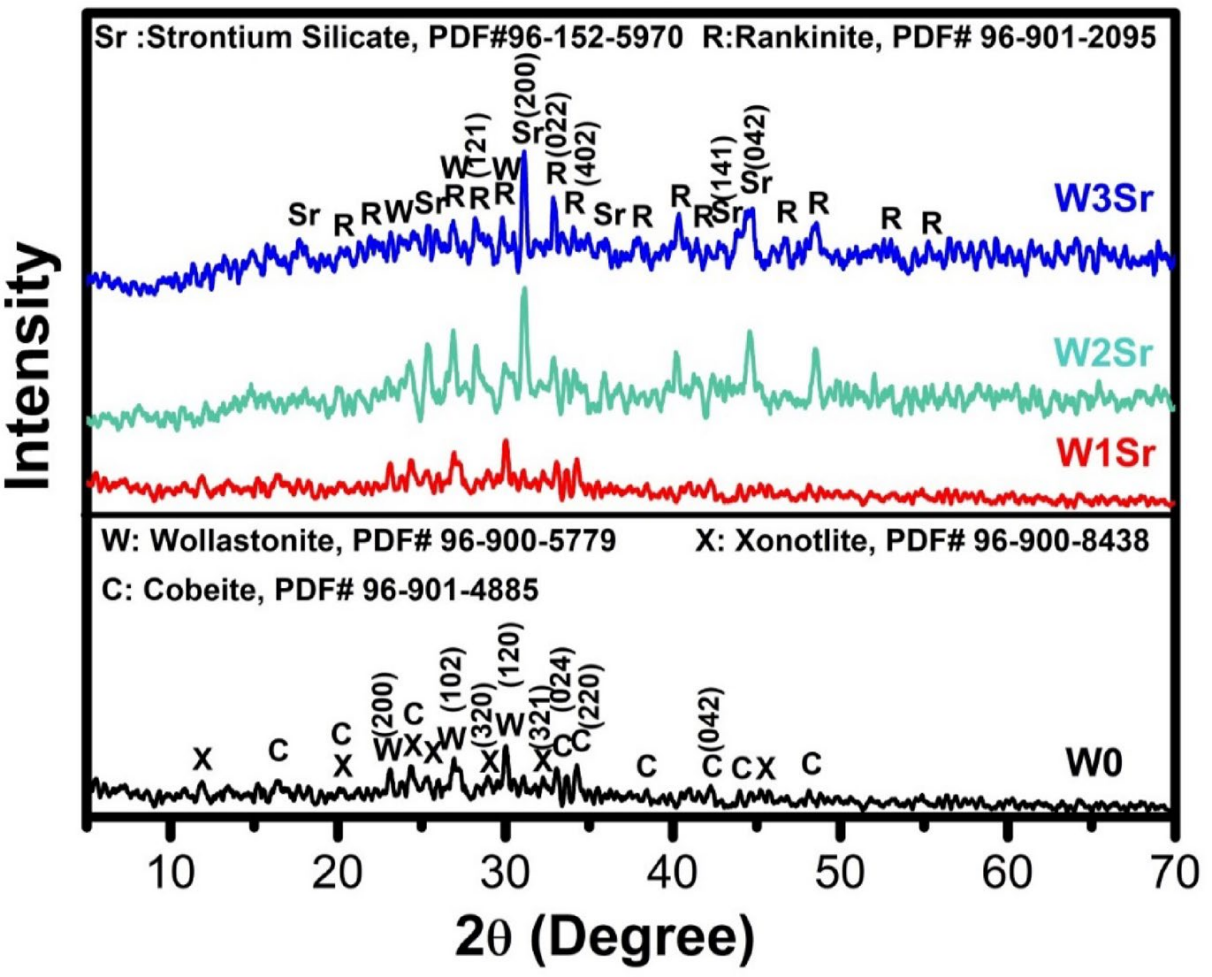
XRD patterns of glass samples heat-treated at 1000℃ for 2 h.

XRD analysis of glass samples heat-treated at 1000 °C for 2 h (Fig. 2) identifies the resulting crystalline phases. The base composition (W0) primarily crystallized as wollastonite (CaSiO₃), with xonotlite (Ca_6_Si_6_O_19_) and combeite (Na_4_Ca_4_Si_6_O_18_) present as secondary phases. The presence of xonotlite and combeite as secondary crystalline phases is attributed to the inherent glass chemistry and crystallization behavior of the CaO–SiO₂–Na₂O system. The high CaO/SiO₂ ratio promotes multiple calcium silicate polymorphs, enabling xonotlite crystallization^22^, while Na₂O facilitates network depolymerization and phase separation, leading to combeite formation^36^. In contrast, the SrO-substituted samples (W1Sr, W2Sr, W3Sr) crystallized with rankinite (Ca_2_Si_2_O_7_) as the primary phase, accompanied by strontium silicate (SrSiO_3_). Our findings are consistent with earlier studies by *Wu and Zreiqat*^9^ and by *Salman et al.*^37^, who reported that introducing strontium into the CaSiO₃ structure leads to an enlargement of the crystal size. This growth in crystal dimensions may facilitate atomic diffusion and contribute to the promotion of phase transitions. Additionally, Na_2_O acts as a network modifier, depolymerizing the silicate network by increasing non-bridging oxygens, which leads to reduced melt viscosity and enhanced atomic mobility during melt quenching and crystallization. This reduction in viscosity can facilitate nucleation and subsequent crystallite growth, acting synergistically with Sr incorporation. Similar combined effects of modifier cations and network depolymerization on crystallization behavior have been widely reported in bioactive and silicate glass systems^38^. Therefore, the observed crystallite growth is more appropriately attributed to the combined influence of Sr ionic size, Na₂O-induced depolymerization, and melt viscosity changes.

**Fig. 3:**
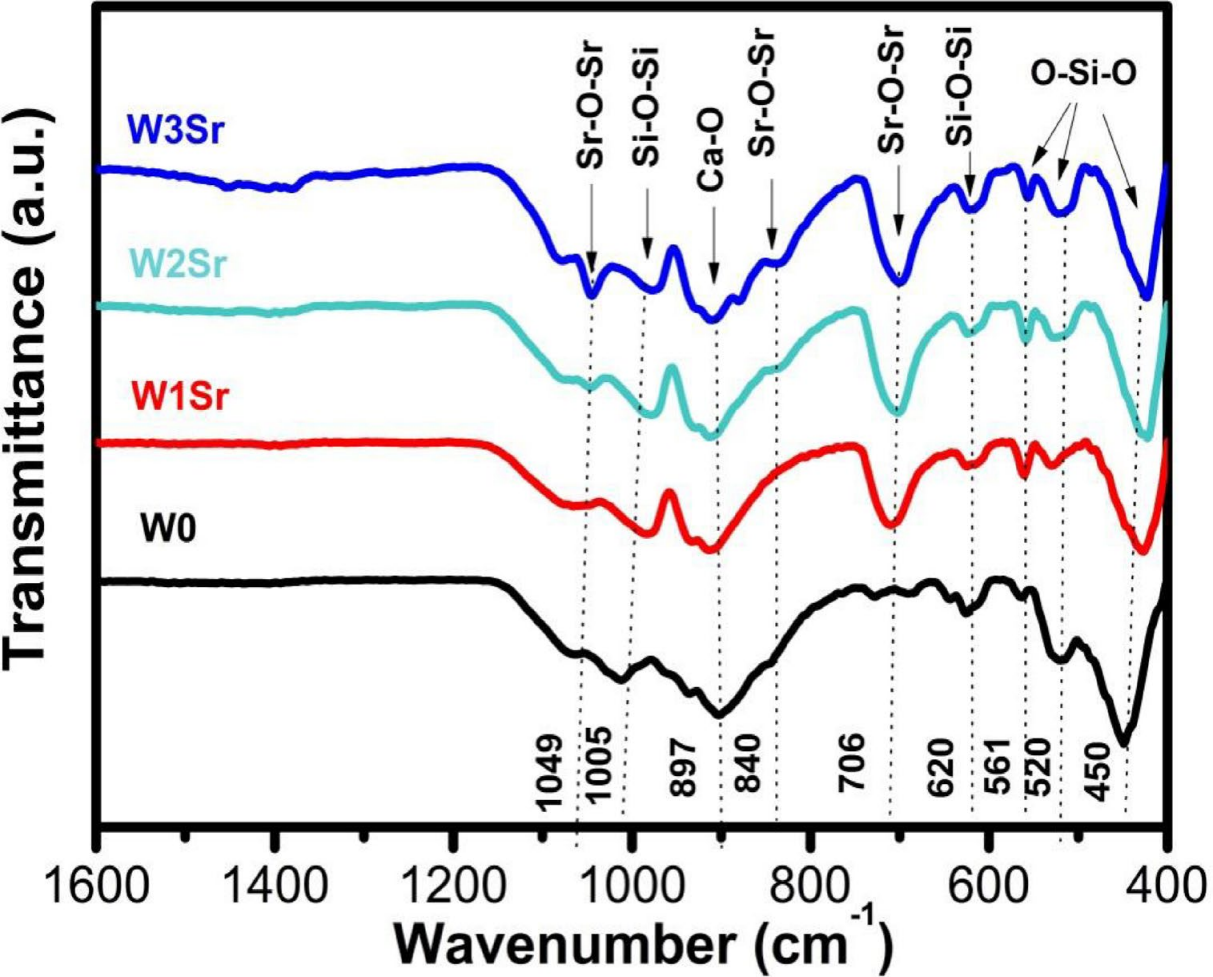
FTIR spectra of the glass samples heat-treated at 1000℃/2 h.

FTIR spectra of the synthesized powder samples, recorded in the range of 400–1600 cm^-1^, are presented in Fig. 3. The spectrum of pure wollastonite (W0), exhibits characteristic peaks at 450, 520, and 561 cm^-1^, attributed to O–Si–O bending vibrations associated with SiO₂ formation^8,39,40^. Additional peaks at 620 and 1005 cm^-1^ correspond to the asymmetric stretching vibrations of Si–O–Si in SiO₄ tetrahedra^40^. A distinct band at ~ 840 cm^-1^ in the pure wollastonite (W0) is specifically attributed to the antisymmetric stretching vibrations of Si–O–Si^41^, while the peak at 897 cm^-1^ and the broad band spanning 1400–1500 cm^-1^ are assigned to Ca–O bonding^8^. For SrO-substituted samples (W1Sr, W2Sr, W3Sr), new vibrational modes emerge at ~ 706 and 1049 cm^-1^ (Sr–O–Sr bonds) and a weak absorption band at ~ 840 cm⁻¹, confirming SrO incorporation^41–43^. The intensity of these IR bands increases progressively with higher SrO/CaO substitution ratios.

**Fig. 4:**
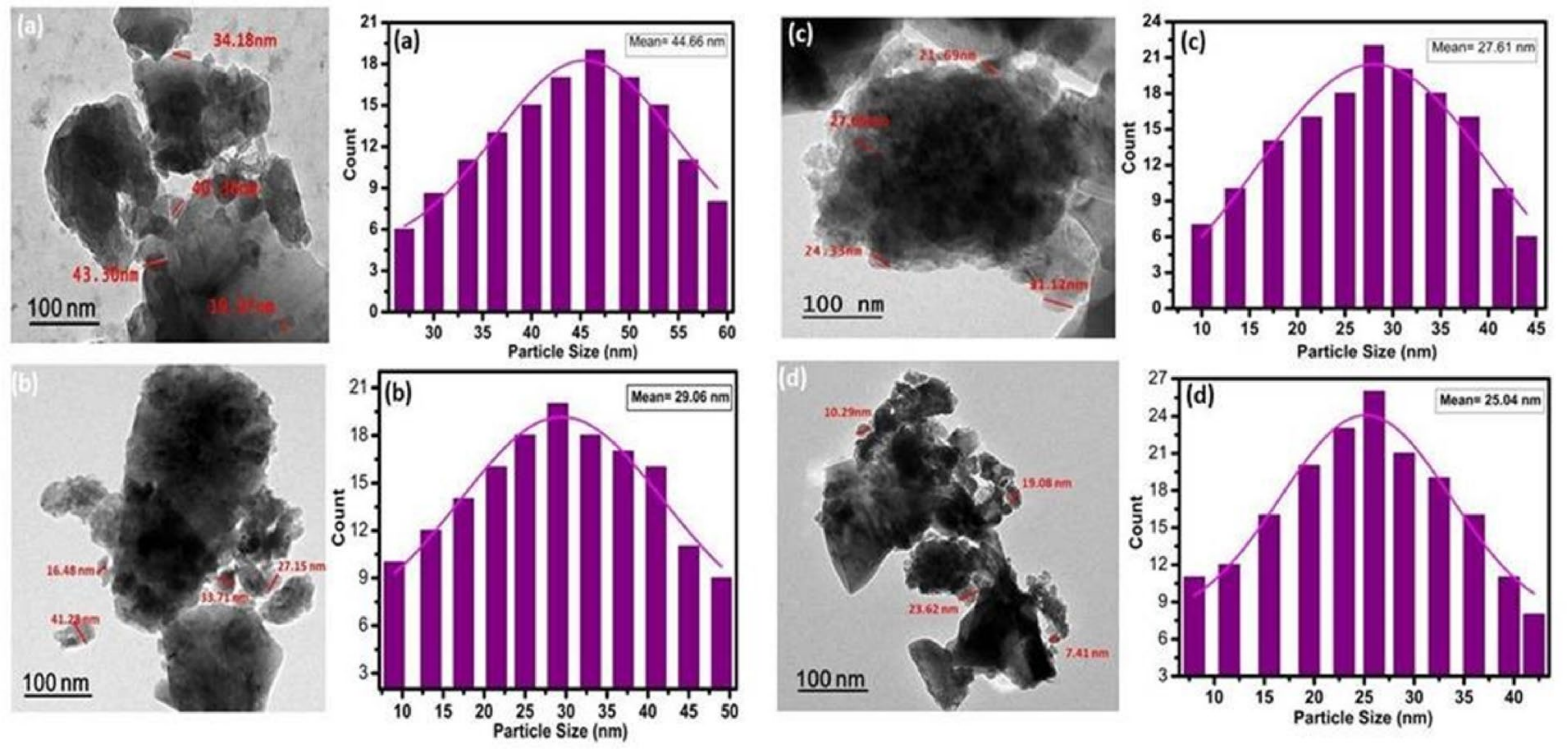
HR-TEM images and corresponding particle size distribution histograms for samples (**a**) W0, (**b**) W1Sr, (**c**) W2Sr, and (**d**) W3Sr.

HR-TEM images of the heat-treated samples (1000 °C for 2 h) are presented in Fig. 4a–d. As the SrO content increases, the particle size distribution becomes narrower and shifts toward smaller sizes. Specifically, crystallite sizes decrease from 19.97 to 43.30 nm in the base glass-ceramic (W0) to 9.27–21.83 nm in the W3Sr sample, demonstrating a clear inverse correlation between the SrO/CaO substitution ratio and crystallite size. This refinement in particle size is attributed to the doping effect of Sr²⁺ ions, which act as crystal growth inhibitors by inducing lattice strain and hindering atomic attachment at the growing crystal surface. This inhibition of crystal growth simultaneously promotes higher nucleation density, resulting in a finer-grained microstructure^45^.

**Fig. 5:**
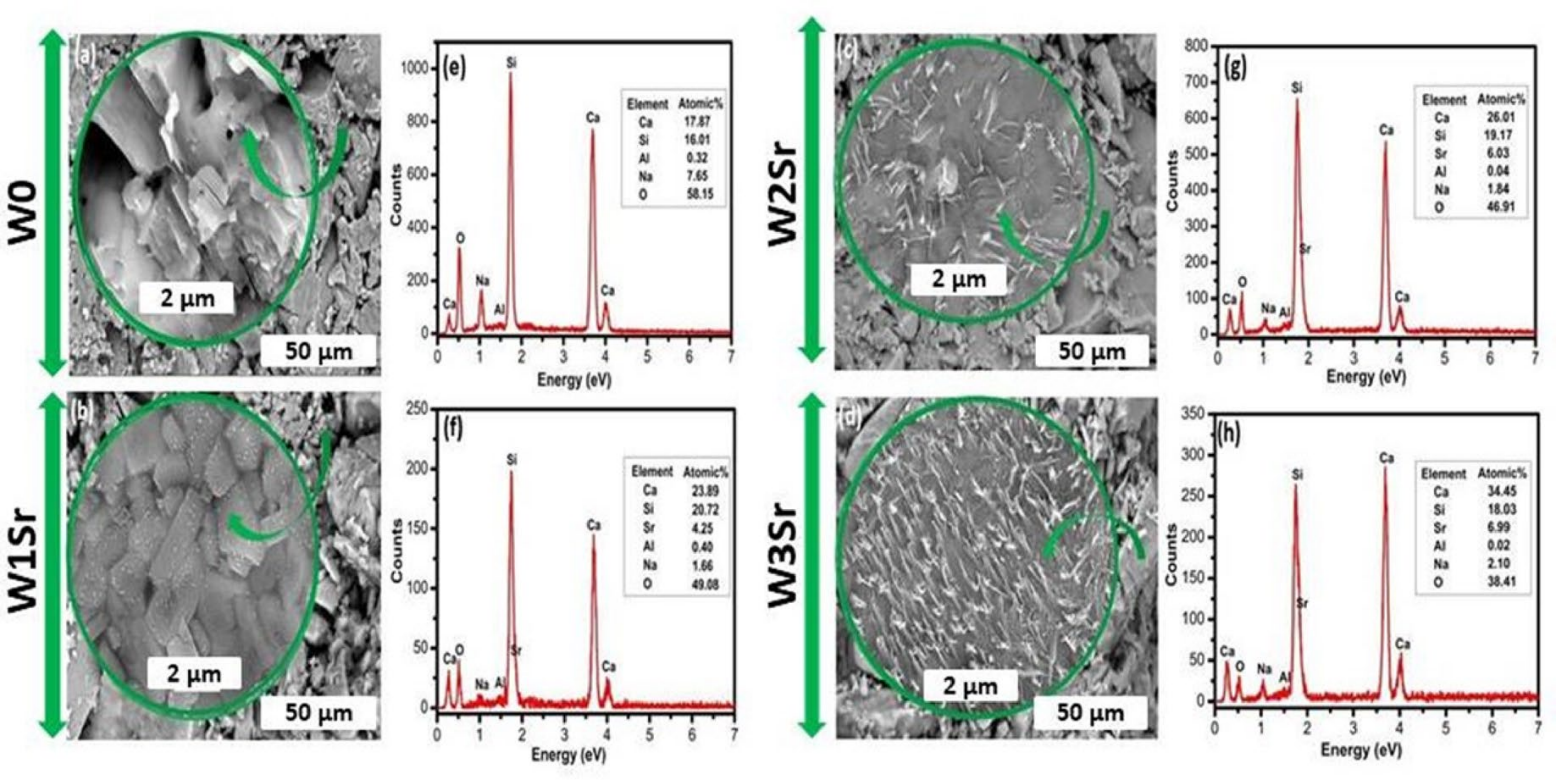
FE-SEM images (**a**–**d**) and corresponding EDX microanalysis (**e**–**h**) of Sr-doped wollastonite samples (W0, W1Sr, W2Sr, W3Sr) after heat treatment at 1000 °C for 2 h.

FE-SEM analysis (Fig. 5) reveals a clear evolution in microstructure with increasing SrO substitution. The base sample (W0), heat-treated at 1000 °C for 2 h, displays irregular, poorly sorted submicron grains and lathe-shaped nanoparticles, indicative of uncontrolled crystal growth (Fig. 5a). In contrast, the Sr-substituted samples (W1Sr, W2Sr, W3Sr) show progressively more defined and distinct nanoscale morphologies. At lower Sr concentrations, W1Sr (Fig. 5b) predominantly consists of roughly spherical nanoparticles, suggesting that nucleation is favored over growth, with limited Sr incorporation promoting isotropic crystal development. With increasing Sr content, the microstructure of W2Sr evolves into elongated, needle-like nanostructures (Fig. 5c). This change in morphology indicates a shift in crystal growth kinetics and surface energy, favoring anisotropic growth along specific crystallographic directions. Further increasing the SrO content in W3Sr (Fig. 5d) results in spindle-shaped nanoparticles elongated yet wider at the center likely due to increased lattice strain or modified surface chemistry. This morphology suggests a dynamic balance between nucleation and directional growth rates influenced by the higher Sr incorporation^42^. EDX microanalysis confirmed the expected elemental composition across all samples, with trace amounts of aluminum attributed to minor impurities from the raw materials.

### In vitro bioactivity of the produced glass-ceramics

The bioactivity and biocompatibility of sintered glass-ceramics were assessed through immersion in SBF for 28 days. Surface properties of the processed samples were analyzed via XRD, FTIR, and FE-SEM/EDS. Mechanical and physical characterization included measurements of compressive strength, bulk density, apparent porosity, and degradation behavior.

#### X-ray diffraction analysis

**Fig. 6:**
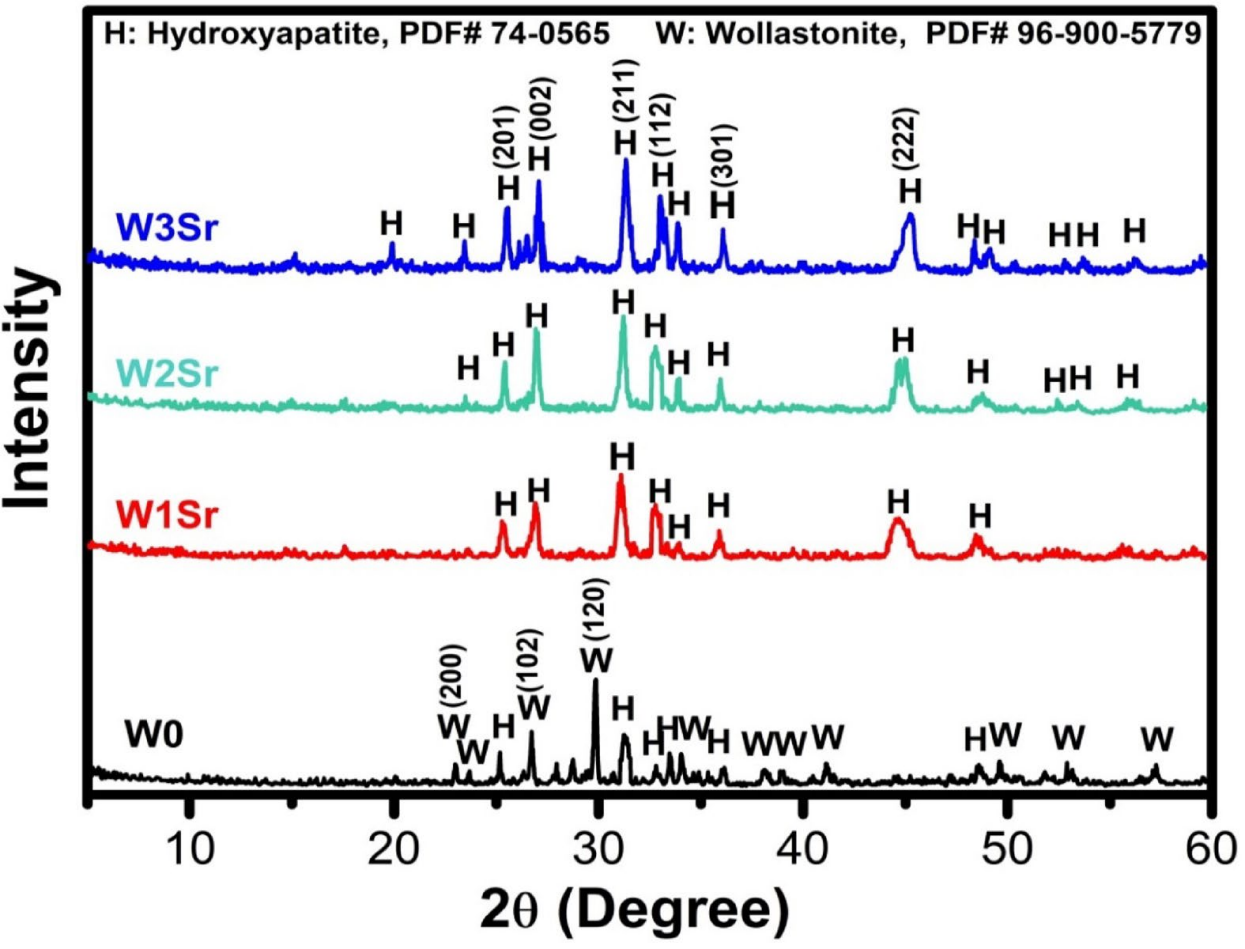
XRD patterns of Sr-doped wollastonite samples following 28 days of immersion in SBF.

Following 28 days of immersion in SBF, X-ray diffraction (XRD) analysis of Sr-containing wollastonite ceramics (Fig. 6) revealed diminished intensity in the original wollastonite phase peaks (W0) and the emergence of new peaks consistent with hydroxyapatite (HA; JCPDS #74–0565). In Sr-substituted samples (W1Sr, W2Sr, W3Sr), strontium silicate peaks were completely absent, replaced by distinct HA reflections. These findings confirm the formation of a hydroxyapatite surface layer on all ceramics upon immersion in SBF. The presence of rankinite (Ca₃Si₂O₇) and SrSiO₃ phases present in Sr-substituted samples contribute differently to bioactivity and degradation behavior. Due to rankinite contributes sustained silicate and calcium release supporting long-term bioactivity^46^, while SrSiO₃ provides a faster release of Sr²⁺ and Si ions that can enhance early surface reactions and apatite nucleation^47^. As a result, the overall bioactivity and degradation behavior of the material reflect the combined contributions of both phases rather than a single phase alone. Notably, pure wollastonite (W0) exhibited weaker HA peak intensity compared to Sr-doped variants, with the highest Sr concentration (W3Sr) showing the most pronounced HA crystallization^42^.

#### FTIR spectroscopy

**Fig. 7:**
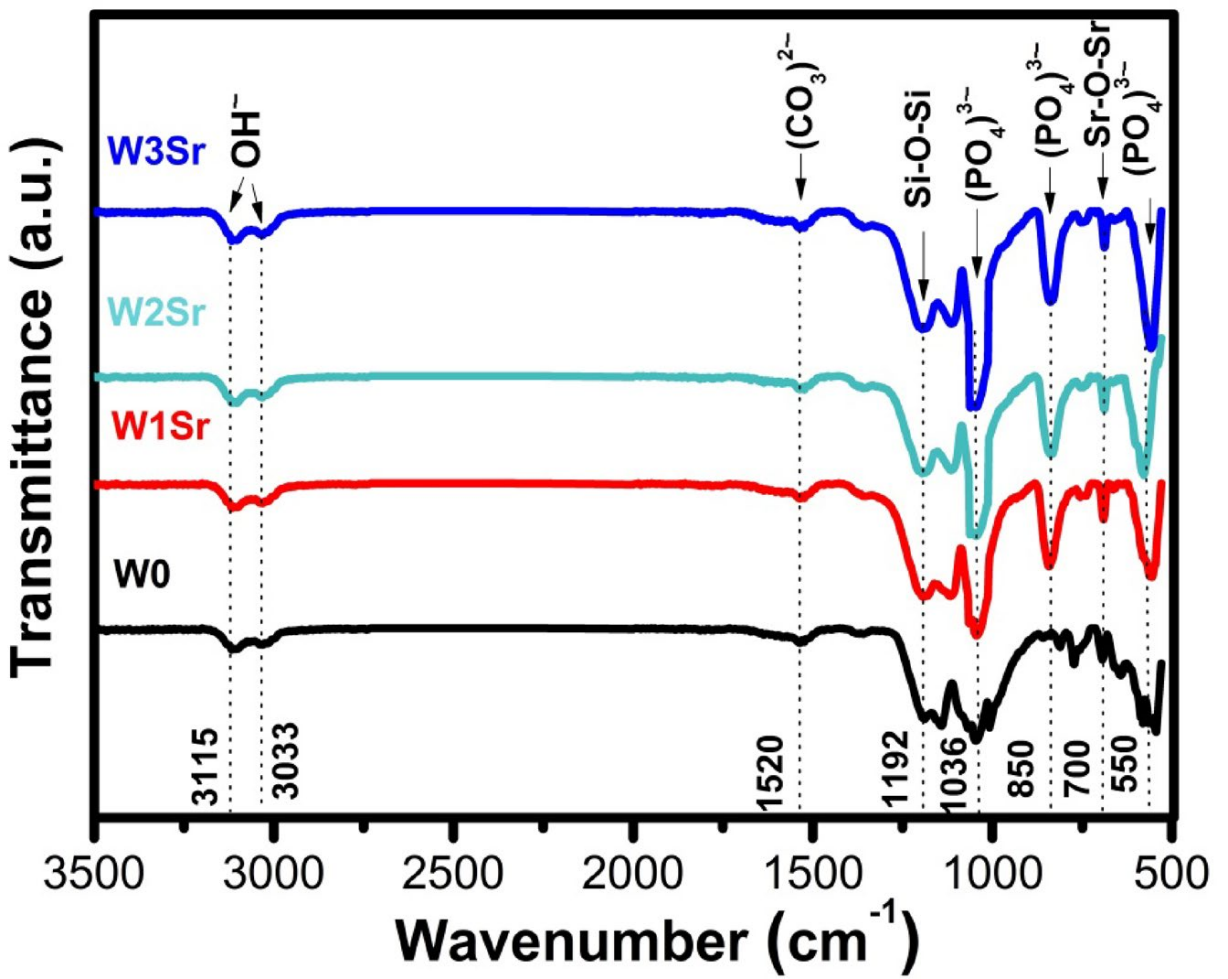
FTIR spectra of wollastonite containing Sr after soaking in SBF for 28 days.

Figure 7 presents the FTIR spectra of Sr-doped wollastonite ceramics after 28 days of immersion in SBF. Characteristic phosphate (PO₄³⁻) vibrations are observed at around 550 cm⁻¹, corresponding to ν₄ bending, and at approximately 1036 cm⁻¹, attributed to ν₃ stretching modes, confirming the formation of a calcium phosphate phase^34,35^. In addition, the presence of carbonate (CO₃²⁻) bands (Fig. 7) in the region of ~ 1500 cm⁻¹ provides evidence for the development of a carbonate-containing apatite layer, indicative of bioactive behavior^21,48^. More specifically, bands located near ~ 1520 cm⁻¹ (ν₃ CO₃²⁻) and ~ 850–870 cm⁻¹ (ν₂ CO₃²⁻) are consistent with B-type carbonate substitution, where carbonate ions replace phosphate groups within the apatite lattice. The absence of features typically associated with A-type substitution, such as distinct bands near ~ 1545 cm⁻¹ and notable changes in the OH⁻ stretching region, further supports this assignment. Moreover, the progressive increase in carbonate band intensity relative to phosphate bands with increasing Sr content suggests a higher degree of B-type carbonation. This type of carbonated apatite closely resembles natural bone mineral, indicating that the formed apatite layer is predominantly B-type carbonated apatite and thus biologically favorable for bone-like mineralization^5,8,41,49^. So the relative intensities observed in Fig. 7 strongly indicate that the apatite layer is predominantly B-type carbonated apatite, which is considered more biologically relevant for bone-like mineralization. Additional features include O–H stretching at 3033 and 3115 cm⁻¹^50^, Si–O–Si vibrations at 1192 cm^-1^, and a minor Sr–O–Sr band near 700 cm⁻¹, confirming Sr incorporation^40,42^.

#### FE-SEM/EDX analysis

**Fig. 8:**
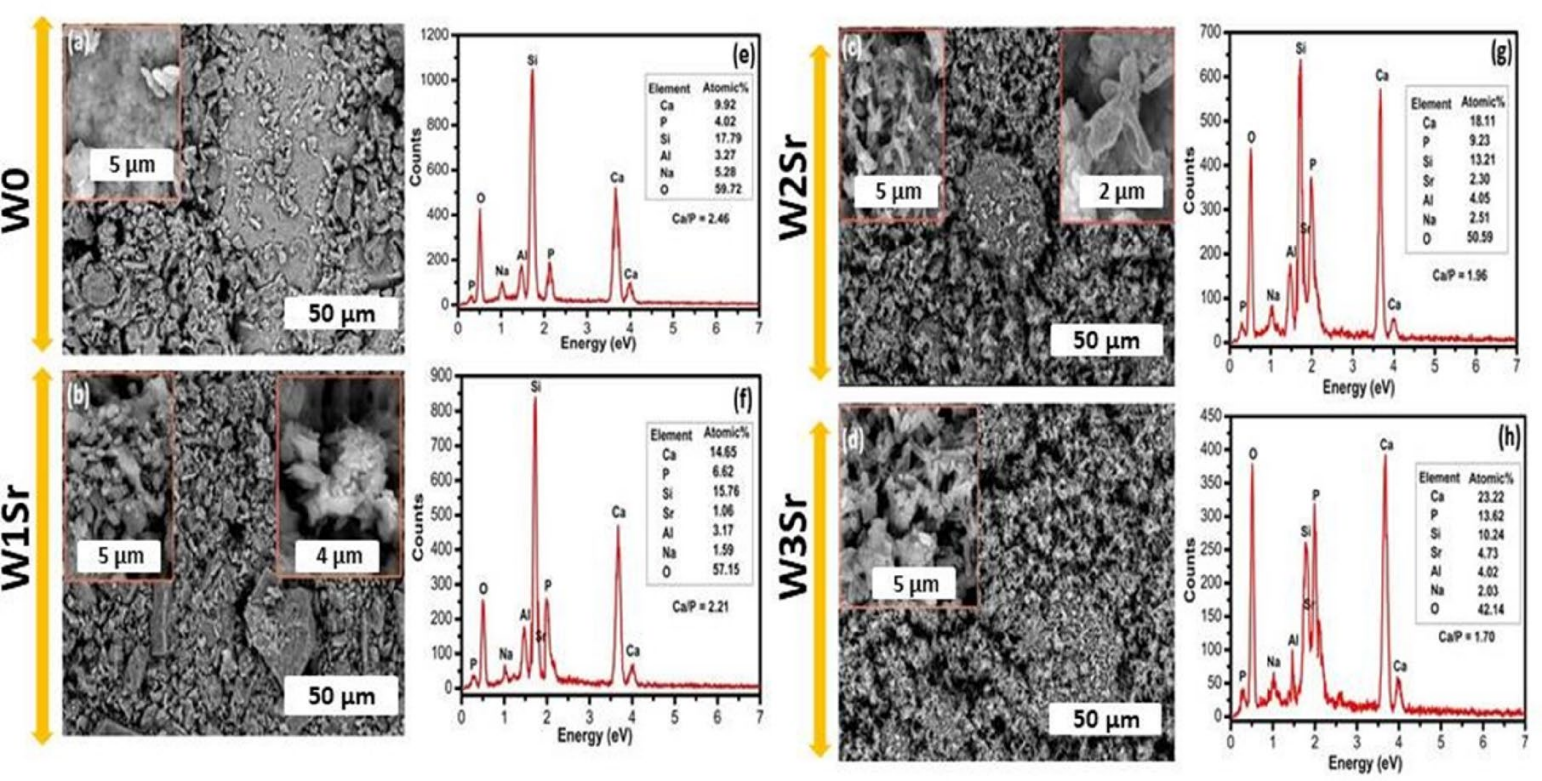
FE-SEM images (**a**–**d**) and EDX spectral data (**e**–**h**) for Sr-doped wollastonite samples (W0, W1Sr, W2Sr, W3Sr) after heat treatment at 1000 °C for 2 h and subsequent 28-day immersion in SBF.

The bioactive potential of Sr-substituted wollastonite ceramics was further validated through elemental composition analysis and surface morphology studies. FE-SEM micrographs (Fig. 8a-d) and corresponding EDX spectra (Fig. 8e-h) of the heat-treated glass-ceramic samples after 28 days of immersion in SBF. All samples were heat-treated at 1000 °C for 2 h prior to testing. As depicted in Fig. 8a–d, hydroxyapatite (HA) layers fully coated all sample surfaces. These morphological changes arise from interactions between the glass-ceramics and ionic species in SBF. Notably, the HA structure transitioned from plate-like multilayers on W0 and W1Sr surfaces to needle-like morphologies in W2Sr and W3Sr as Sr content increased. This evolution underscores the direct influence of Sr substitution levels on HA crystallization behavior, highlighting composition-dependent bioactivity modulation. A secondary observation was the variation in Ca/P atomic ratios across batches, as quantified by EDX spectra Fig. 8e–h. Analysis revealed a progressive decline in the Ca/P ratio from 2.46 (W0) to 1.70 (W3Sr)^6,50^, directly correlating with increased Sr substitution. This reduction aligns the Ca/P ratio of hydroxyapatite (HA) formed on the W3Sr surface with that of natural bone minerals (~ 1.67), a critical factor for promoting bone integration in physiological environments^14^.

The Ca/P ratios in the present study evolve toward ~ 1.67, closely matching the stoichiometric value of hydroxyapatite and aligning well with recent literature^39,40^. In particular, *Kumar and Shikha*^51^ reported a Ca/P ratio of 1.66 for undoped samples and a (Ca + Co)/P ratio of 1.56 for Co-doped systems, both within the natural bone composition range and associated with good biocompatibility. Similarly, the Ca/P values obtained in our work indicate the formation of bone-like apatite, with Sr incorporation causing only minor, biologically acceptable deviations. This comparison confirms that the apatite layers formed on our materials are compositionally comparable to established bioactive ceramic and doped hydroxyapatite systems reported in the literature. Among the tested compositions, the W3Sr batch (0.5 wt% SrO₂) demonstrated optimal stability and bioactivity, mirroring the mineral phase of native bone. These findings underscore its potential for biomedical applications, warranting further validation through targeted in vivo studies.

**Fig. 9:**
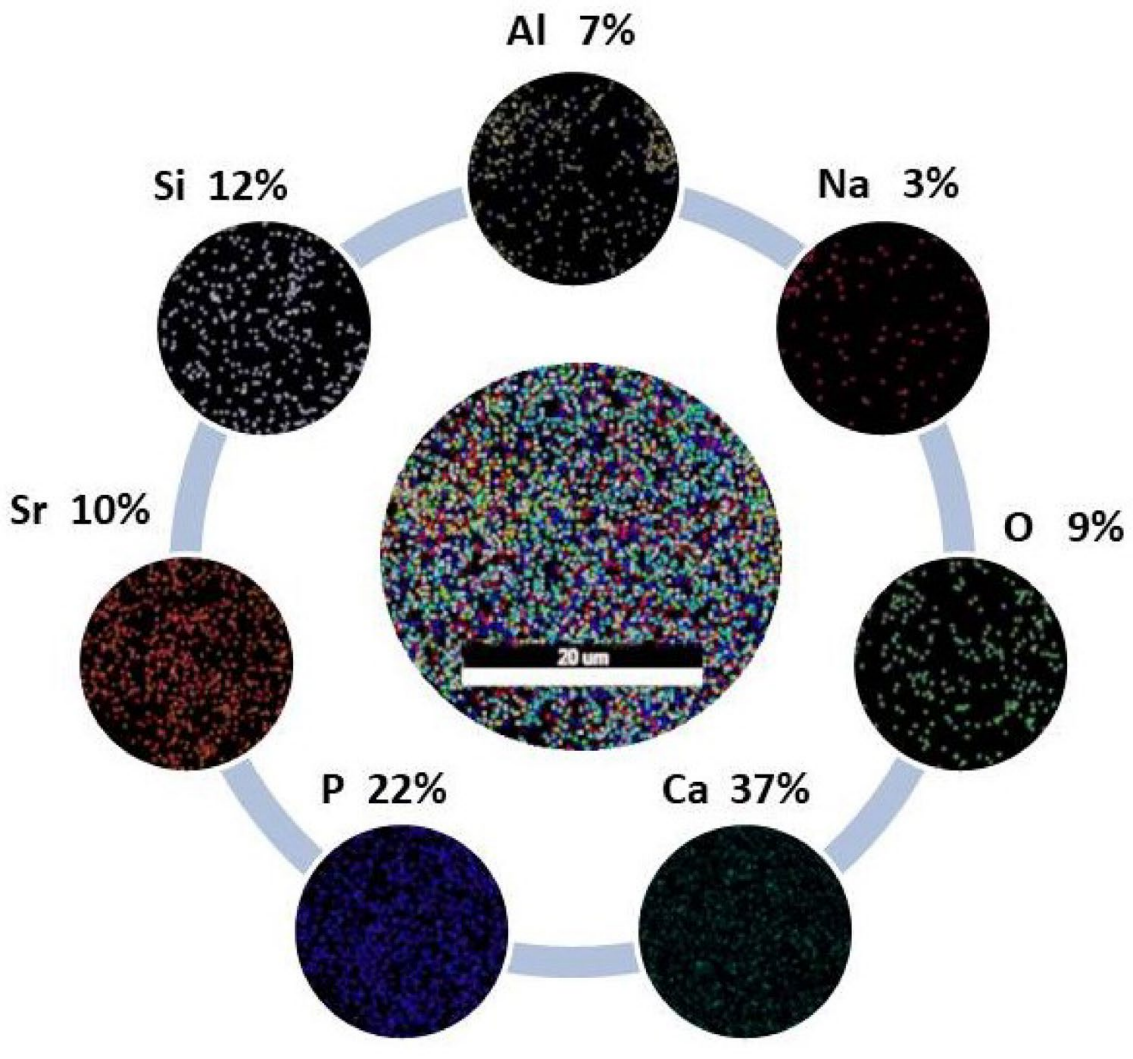
Map photograph of the best group W3Sr after soaking for 28 days in SBF.

As shown in Fig. 9, elemental mapping photograph of the optimized wollastonite formulation (W3Sr, 0.5 wt% SrO₂) visually confirms the formation of a hydroxyapatite layer, validating the quantitative EDS results presented in Fig. 8h. The map shows that W3Sr is the best composition because its Sr-induced fine microstructure optimizes the formation of a thick, uniform, and therapeutic apatite layer, making it a highly promising candidate for bone graft substitutes and coatings for orthopedic implants.

#### Physico-mechanical behavior

**Fig. 10:**
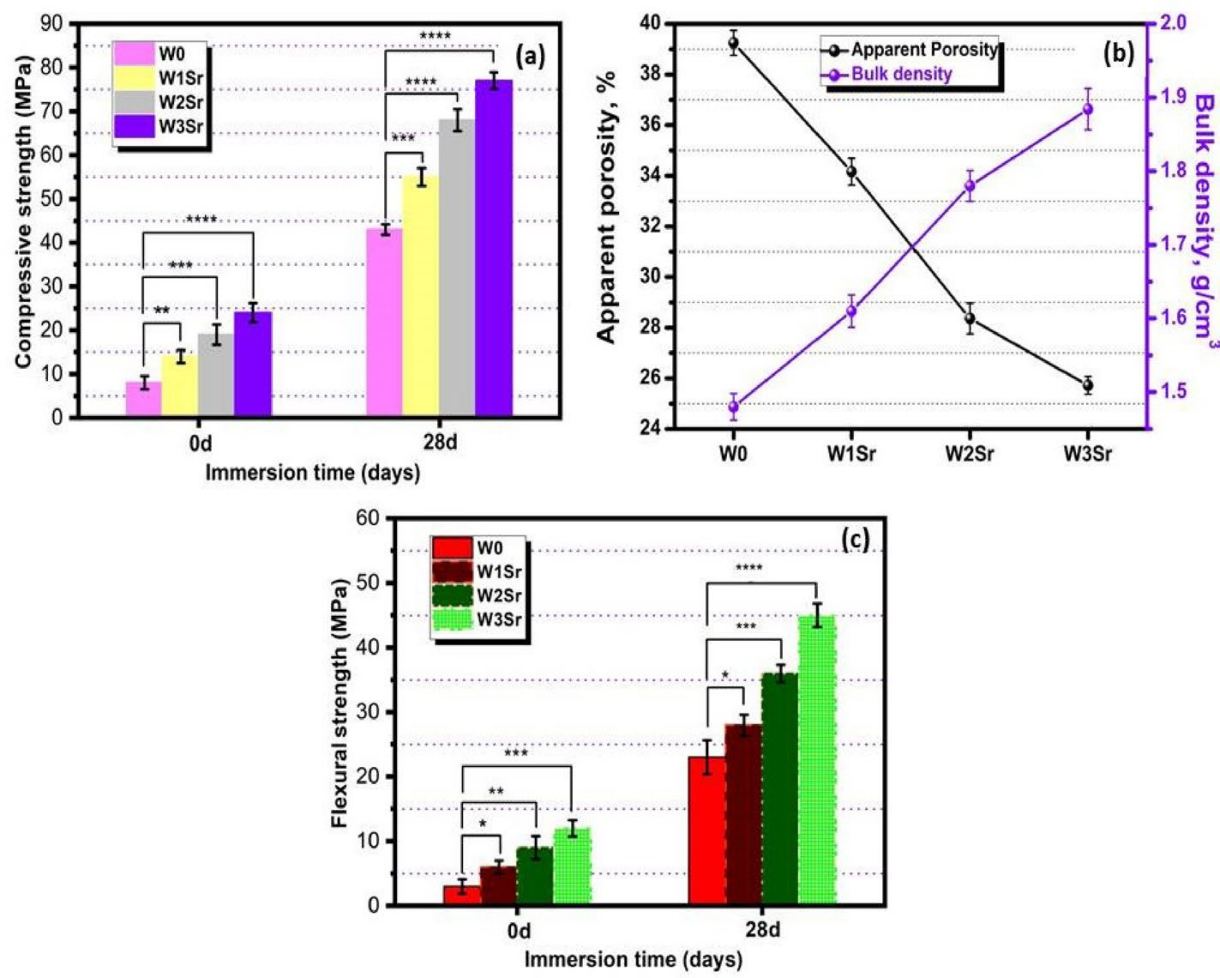
Impact of 28-days SBF immersion (37 °C) on the physico-mechanical performance of Sr-doped wollastonite ceramics. Data are presented as mean ± SD from triplicate experiments. Significant differences compared to the undoped control (W0) are marked as follows: *p ≤ 0.05 **p ≤ 0.01, ***p ≤ 0.001, ****p ≤ 0.0001.

Figure 10a demonstrates the compressive strength evolution of Sr-doped wollastonite ceramics before and after 28-day SBF immersion. Initially, Sr incorporation marginally enhanced strength relative to pure wollastonite, attributable to strontium’s larger ionic radius compared to calcium promoting unit cell expansion and densification during sintering. These changes improve molecular transport and strengthen the ceramic matrix, ultimately boosting mechanical performance^4,52^. Following immersion, all compositions exhibited substantially increased strength, with values rising progressively with Sr content: W0 (43 ± 1.2 MPa), W1Sr (55 ± 2.05 MPa), W2Sr (68 ± 2.3 MPa), and W3Sr (77 ± 1.9 MPa). This enhancement is attributed to two synergistic mechanisms: the formation of a reinforcing apatite layer facilitated by wollastonite-derived SiO₂-rich gel layer^40^, and improved interfacial bonding due to Sr²⁺, lower electronegativity, which stabilizes the Si–O–Sr bonds and promotes Ca²⁺ substitution in the apatite structure^22,53,54^. Hence it may be concluded that the strontium doping significantly enhances the compressive strength of ceramics, both pre- and post-immersion in SBF, due to its dual role in promoting material densification and forming a reinforced bone-like apatite surface layer. In this study, the compressive strength of the Sr-substituted wollastonite ceramics increased with Sr content, approaching values that are broadly comparable with those of natural bone tissue. These comparisons indicate that the tested ceramics fall within or below the lower range of cortical bone and well above typical trabecular bone strength, suggesting suitability for non-load-bearing or moderate load-bearing orthopedic applications such as scaffold materials, bone fillers, or coatings^55^. Importantly, the improved compressive strength and enhanced in vitro bioactivity supports their potential for clinical use where biological performance and gradual load transfer to regenerating bone are critical.

Figure 10b shows that after 28 days in SBF, higher Sr content in wollastonite ceramics systematically increases bulk density and reduces porosity. Density values rose from 1.48 ± 0.018 g/cm³ (W0) to 1.88 ± 0.028 g/cm³ (W3Sr), attributed to hydroxyapatite surface precipitation forming a denser hydrated structure, in that way elevating bulk density and suppressing porosity^8,56,57^. Elevating strontium content produced a definitive trend toward greater structural integrity: bulk density increased markedly while apparent porosity decreased, directly accounting for the enhanced compressive strength. This improvement stems from the role of Sr²⁺ ions in promoting superior sintering and more efficient network packing, yielding a denser, more homogeneous microstructure^18^. However, the enhanced bioactivity of these compositions transcends these physical advantages. Sr²⁺ ions actively mediate biological outcomes by stimulating osteoblast proliferation, inhibiting osteoclast activity, and accelerating the deposition of a bone-like apatite layer. Thus, the Sr-substituted ceramics achieve a synergistic balance leveraging microstructural densification for mechanical robustness while concurrently utilizing controlled porosity and the ionic release of biologically active Sr²⁺ to significantly elevate bioactivity^18,42^.

Based on the observed compressive strength trends, the flexural strength (Fig. 10c) increases with increasing Sr content, following the order W3Sr > W2Sr > W1Sr > W0. This behavior is primarily attributed to Sr incorporation, which reduces porosity and increases bulk density, thereby decreasing the number of surface and internal defects that act as stress concentrators during bending. In addition, Sr-induced microstructural refinement enhances grain-to-grain contact and intergranular bonding, improving load transfer under flexural loading. The further increase in flexural strength observed after 28 days of SBF immersion can be associated with apatite precipitation and partial pore filling, which densify the structure and reinforce the surface layer. Overall, Sr addition effectively enhances the flexural performance of the ceramics while maintaining their bioactive characteristics^42^.

#### Degradation studies

**Fig. 11:**
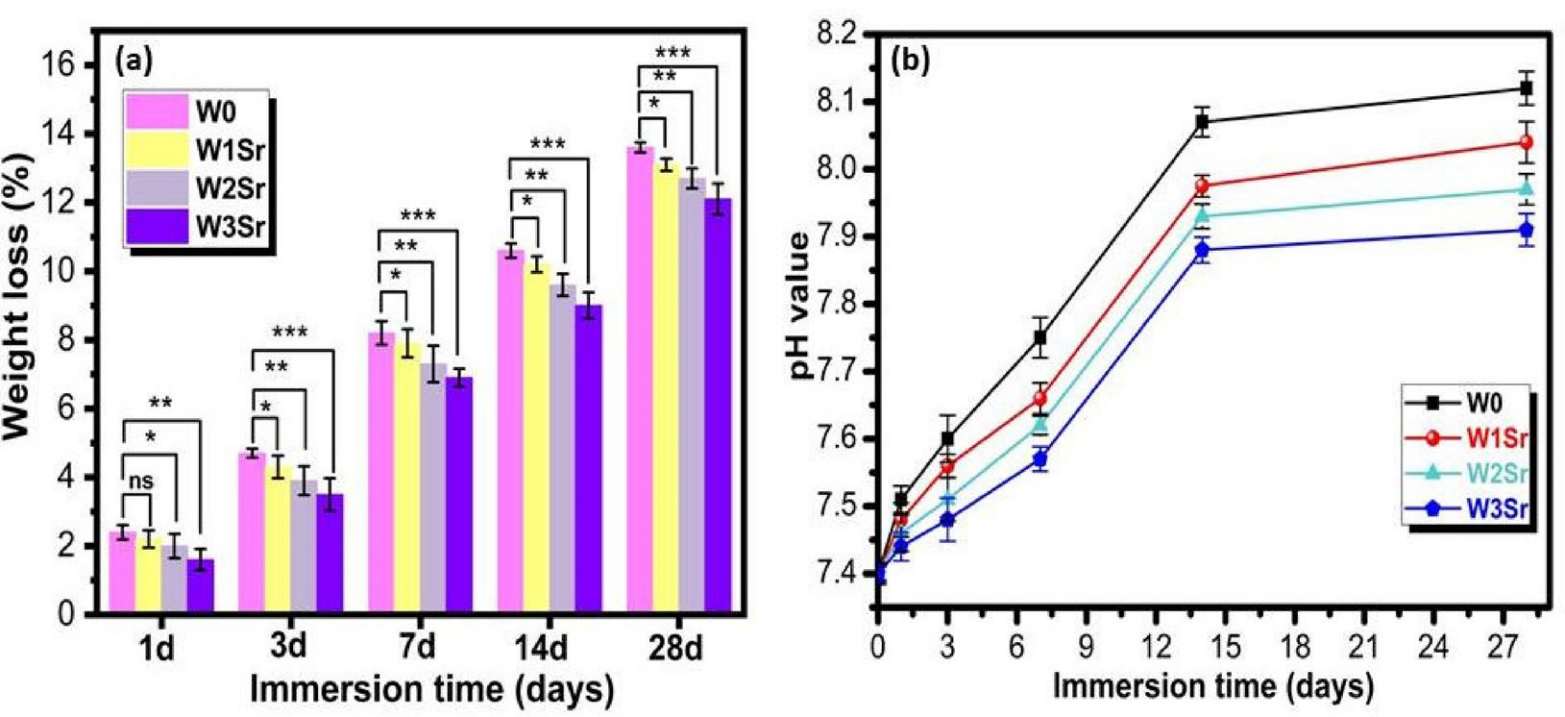
Degradation behavior of Sr-doped wollastonite ceramics during a 28-days immersion in SBF (37 °C), as shown by (**a**) weight loss and (**b**) solution pH value. Data represent the mean ± SD of three independent experiments. Statistical significance relative to the undoped control (W0) is indicated: *p ≤ 0.05, **p ≤ 0.01, ***p ≤ 0.001; ns = not significant (*p* > 0.05).

Degradation kinetics, is a vital characteristic for bone graft materials to synchronize with osseous regeneration, were investigated in Sr-doped wollastonite ceramics (Fig. 11a). All specimens underwent gradual degradation in SBF, with undoped wollastonite (W0) exhibiting the highest mass loss (13.6%). A clear inverse correlation emerged between Sr content and degradation rate, culminating in the 0.5% SrO-doped sample (W3Sr) displaying the lowest mass loss (12.1%). This suppression of degradation is attributed to two synergistic mechanisms: first, the substitution of Ca with Sr enhances ceramic densification and reduces open porosity, limiting SBF infiltration and ionic dissolution, thereby slowing material breakdown^8^; second, the larger ionic radius of Sr²⁺ compared to Ca²⁺ induces lattice distortion, which hinders ion diffusion and delays structural disintegration^42^. These results highlight Sr’s capacity to strategically modulate degradation through coupled microstructural and atomic-scale effects, positioning Sr-doped wollastonite as a tunable biomaterial for bone repair applications where controlled resorption is critical. The biodegradation profile of Sr-doped wollastonite ceramics confirms their strategic suitability for bone regeneration. Undoped wollastonite (W0) degraded fastest, while increasing Sr content progressively reduced mass loss, with 0.5% SrO-doped (W3Sr) samples showing the slowest degradation. This behavior results from improved densification, reduced porosity, and lattice distortion caused by Sr²⁺ substitution, which collectively slow SBF infiltration and ion diffusion. Such tunable degradation ensures mechanical support during bone regeneration while allowing bioactive ion release, demonstrating that Sr doping can strategically control resorption rates to match osseous healing requirements^14,51^.

Figure 11b elucidates the pH change of SBF over time when Sr-doped wollastonite ceramics were immersed. All samples caused a rapid pH increase within the first 14 days, followed by stabilization. This trend corresponds to apatite formation and nucleation on the material surface: initial Ca²⁺ release raises the pH via exchange with H⁺/H₃O⁺ ions in the SBF, enabling a silica-rich layer to attract Ca²⁺ and PO₄³⁻ ions that crystallize into apatite (Eqs. 6 and 7). The subsequent pH plateau reflects reduced ion release and acidic effects from the silica layer^1,7^. Notably, Sr-doped samples preserved slightly lower pH levels than pure wollastonite, consistent with their slower dissolution and reduced mass loss as denoted in (Fig. 11a), underscoring Sr’s role in enhancing structural stability^42^.

6 $${\mathrm{Si}} - {\mathrm{O}} - {\mathrm{Ca}} - {\mathrm{O}} - {\text{Si }} + {\text{ 2H}}^{ + } ~ \to {\mathrm{2Si}} - {\text{OH }} + {\text{ Ca}}^{{{\mathrm{2}} + }}$$

7 $${\mathrm{Si}} - {\text{OH }} + {\text{ OH}}^{ - } \to {\mathrm{Si}} - {\mathrm{O}}^{ - } + {\text{ H}}_{{\mathrm{2}}} {\mathrm{O}}$$

**Fig. 12:**
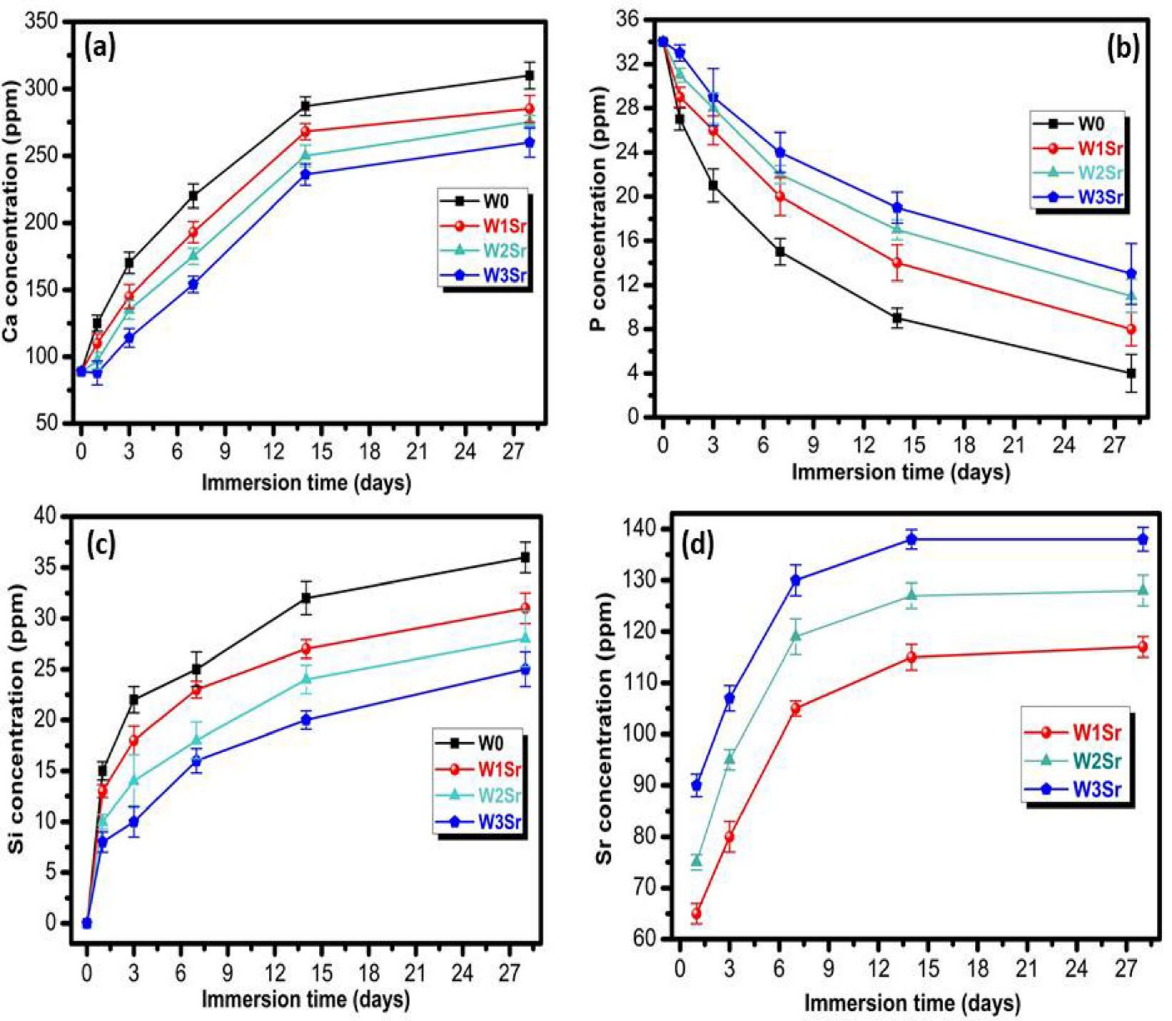
Variation in ion concentration dynamics of Ca, P, Si and Sr in wollastonite ceramics samples after immersion in SBF at 37 °C over 1–28 days. The data are shown as means ± SD of three independent experiments.

The ion release profiles of Sr-doped wollastonite ceramics in SBF over 28 days are shown in Figs. 12a–c. Ca, Si and Sr concentrations increased over time (Figs. 12a, c, and d), while P levels decreased (Fig. 12b). Ca concentration rose sharply within the first 14 days before stabilizing, reaching final values of 310, 285, 277, and 260 ppm for W0, W1Sr, W2Sr, and W3Sr, respectively, and Si concentrations progressively increased to 36, 31, 28, and 25 ppm in the same samples while P levels declined steadily. This steady decrease in phosphorus concentration (Fig. 12b) is a reliable indicator of apatite formation, as phosphate ions are consumed during crystal growth. This decline reflects the material’s bioactivity, with faster depletion indicating more effective apatite formation. It also provides insight into crystallization kinetics and helps identify different stages of the process, such as nucleation, growth, and equilibrium^58^. The bioactive mechanism unfolds through a dynamic interplay of ion exchange and precipitation. Initial dissolution of Ca²⁺ from the ceramic matrix elevates local supersaturation, triggering apatite nucleation that consumes Ca²⁺, PO₄³⁻, and OH⁻ ions. Although incorporated into the growing apatite, the Ca²⁺ dissolution rate outpaces its consumption, leading to a net increase in concentration (Fig. 12a)^51,59^. In wollastonite, the preferential and rapid release of Ca²⁺ over Si⁴⁺ drives an exchange with H⁺ from the solution, generating a hydrated silica layer replete with Si–OH groups. These sites are pivotal, serving as efficient templates for subsequent apatite crystallization^58,60,61^.

Sr²⁺ release from Sr-doped wollastonite ceramics in SBF (Fig. 12d) first rises due to ionic exchange and ceramic matrix disintegration. Long-term immersion causes a silica-rich layer to form and apatite precipitation to start, which lowers and tends to stabilize the release rate. The partial inclusion of Sr²⁺ ions into the growing apatite structure can be responsible for stabilization or a modest reduction at later time points, especially for the W3Sr composition^42^. These ion concentration trends, together with pH changes, corroborate XRD, FTIR, and FE-SEM analyses, confirming apatite layer formation and illustrating the link between dissolution kinetics and surface-mediated mineralization.

#### Antimicrobial assessment

**Fig. 13:**
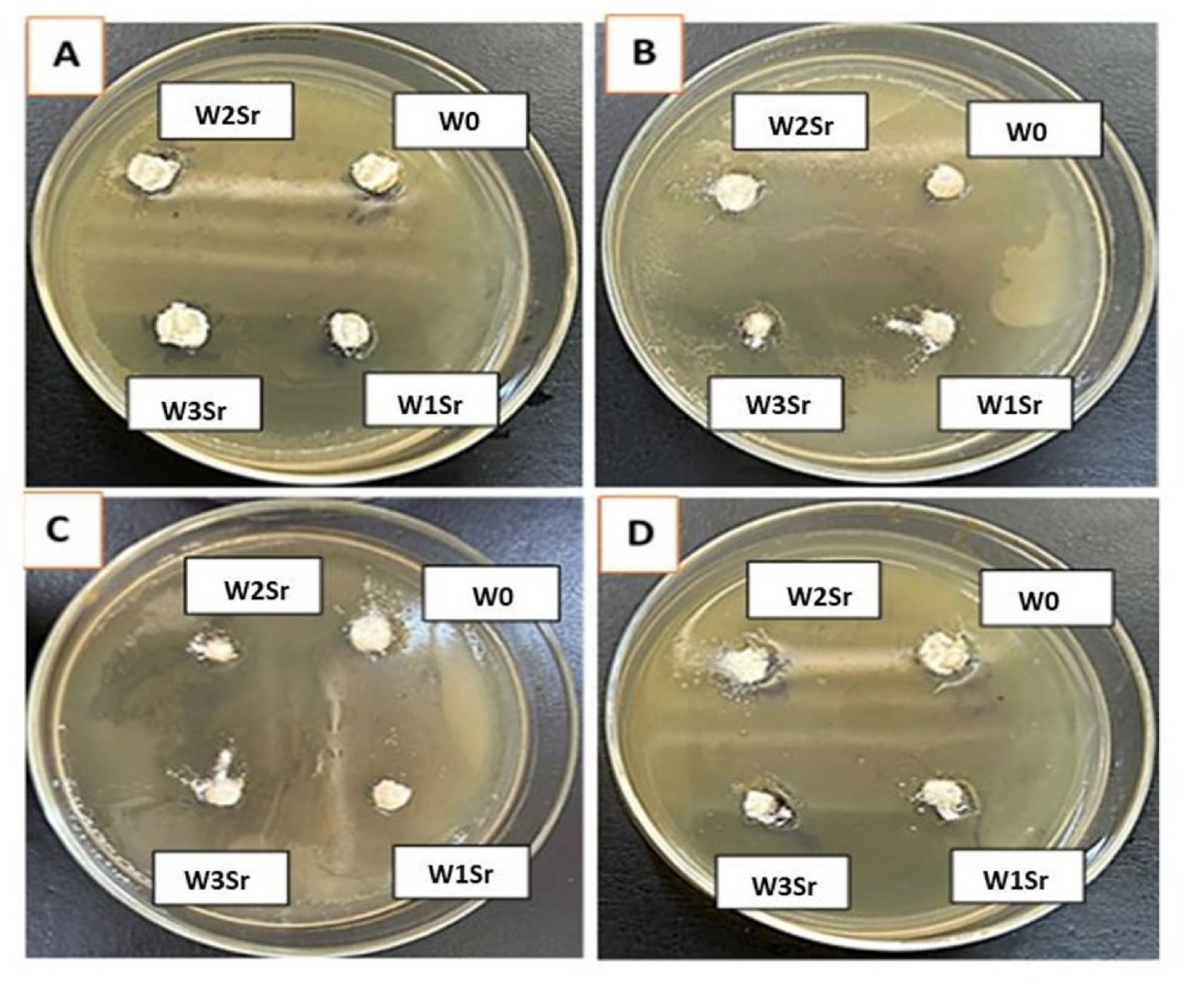
Negative antibacterial effects of glass and glass-ceramic samples against the Gram-negative Bacteria *E. coli* ATCC 25,922 (**A**, **B**) respectively, the Gram-positive Bacteria *S. aureus* ATCC 6538 (**C**, **D**) respectively. 100 mg of each sample placed in ~ 9 mm diameter wells corresponding to Sr²⁺ concentrations of 0.125, 0.25, and 0.50 mg/mL for W1Sr, W2Sr, and W3Sr, respectively.

**Fig. 14:**
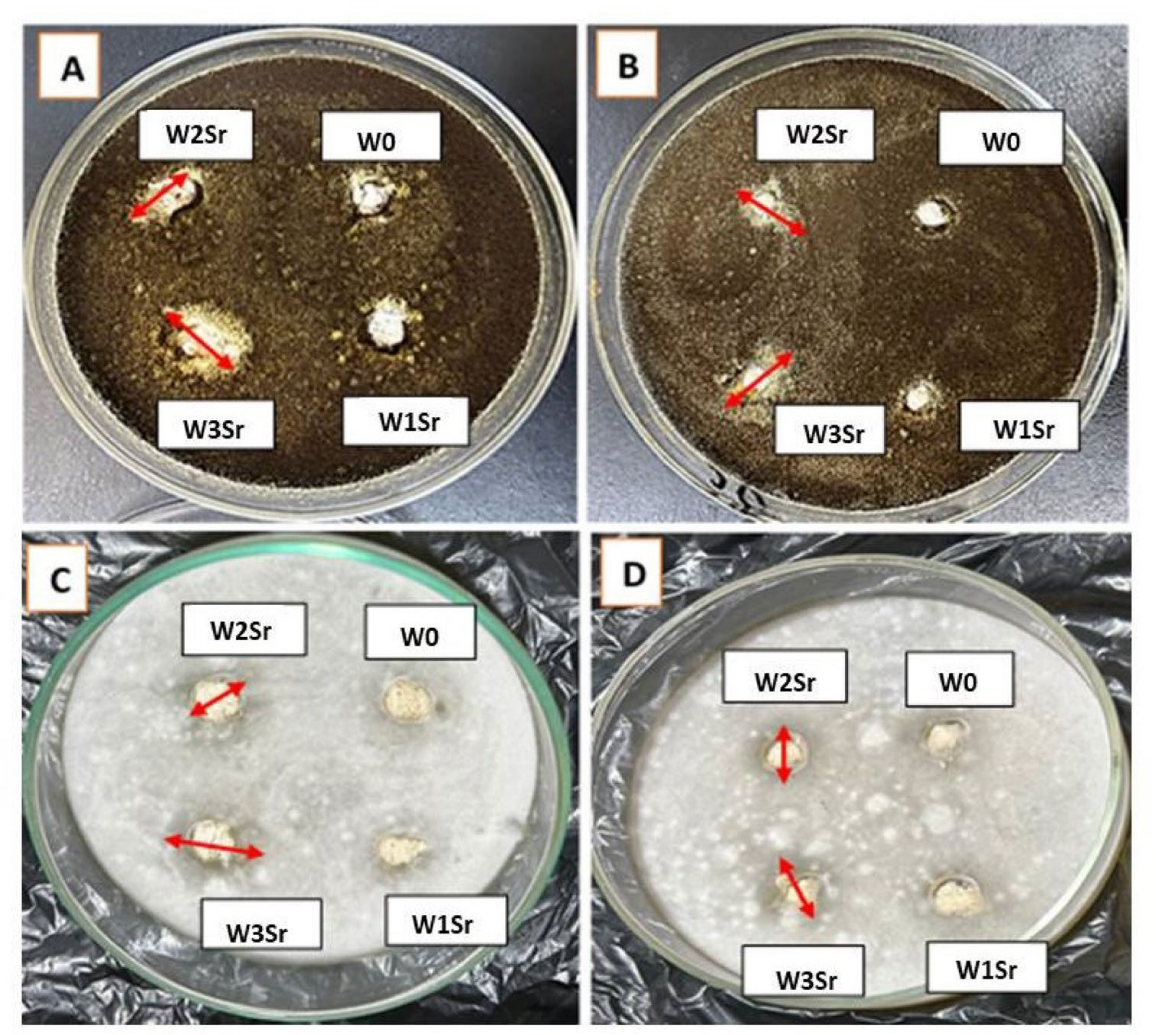
Positive antifungal effects of glass and glass ceramic samples against the filamentous pathogenic fungi: *A. niger* ATCC18666 (**A**, **B**) respectively and *F. solani* NRC18 (**C**, **D**) respectively after 72 h incubation. 100 mg of each sample placed in ~ 9 mm diameter wells corresponding to Sr²⁺ concentrations of 0.125, 0.25, and 0.50 mg/mL for W1Sr, W2Sr, and W3Sr, respectively.

Sr-doped glass and glass-ceramics within Sr concentrations range (0.125–0.50 mg/mL), showed no antibacterial activity against Gram-positive (*S. aureus*) or Gram-negative (*E. coli*) bacteria at any of the four concentrations tested (12, 25, 50, and 100 mg of samples), in addition to the highest dose of 100 mg sample placed in ~ 9 mm diameter wells as shown in Fig. 13. In contrast, a clear dose-dependent antifungal activity was observed at the highest dose of 100 mg sample against *A. niger* and *F. solani* (Fig. 14), with inhibition zone diameters of 11–18 mm (glass- samples) and 10–16 mm (glass ceramic - samples), respectively, at Sr concentrations of 0.25–0.50 mg/mL (Tables 2 and 3). The antifungal efficacy increased with increasing Sr content, reaching a maximum for the pure Sr-glass sample at 0.50 mg/mL (18 mm against *A. niger* and 16 mm against *F. solani*). No inhibition was detected for Sr-free samples, confirming the essential role of Sr in the antifungal response.

The selective antifungal activity, in contrast to the absence of antibacterial inhibition, can be attributed to differences in cell wall structure and physiological characteristics of the target microorganisms. Fungal cells such as *A. niger* and *F. solani* possess a chitin-based cell wall, which is fundamentally different from the peptidoglycan layer of Gram-positive bacteria and the lipopolysaccharide-containing outer membrane of Gram-negative bacteria. These structural differences likely result in variations in Sr²⁺ ion uptake, accumulation, and susceptibility. Moreover, the lack of antibacterial activity suggests that the concentration of Sr²⁺ ions released from the material surface may be insufficient to disrupt bacterial membranes or essential bacterial metabolic processes, while remaining adequate to interfere with fungal metabolism or compromise fungal cell wall integrity^12,51,62^.

**Fig. 15:**
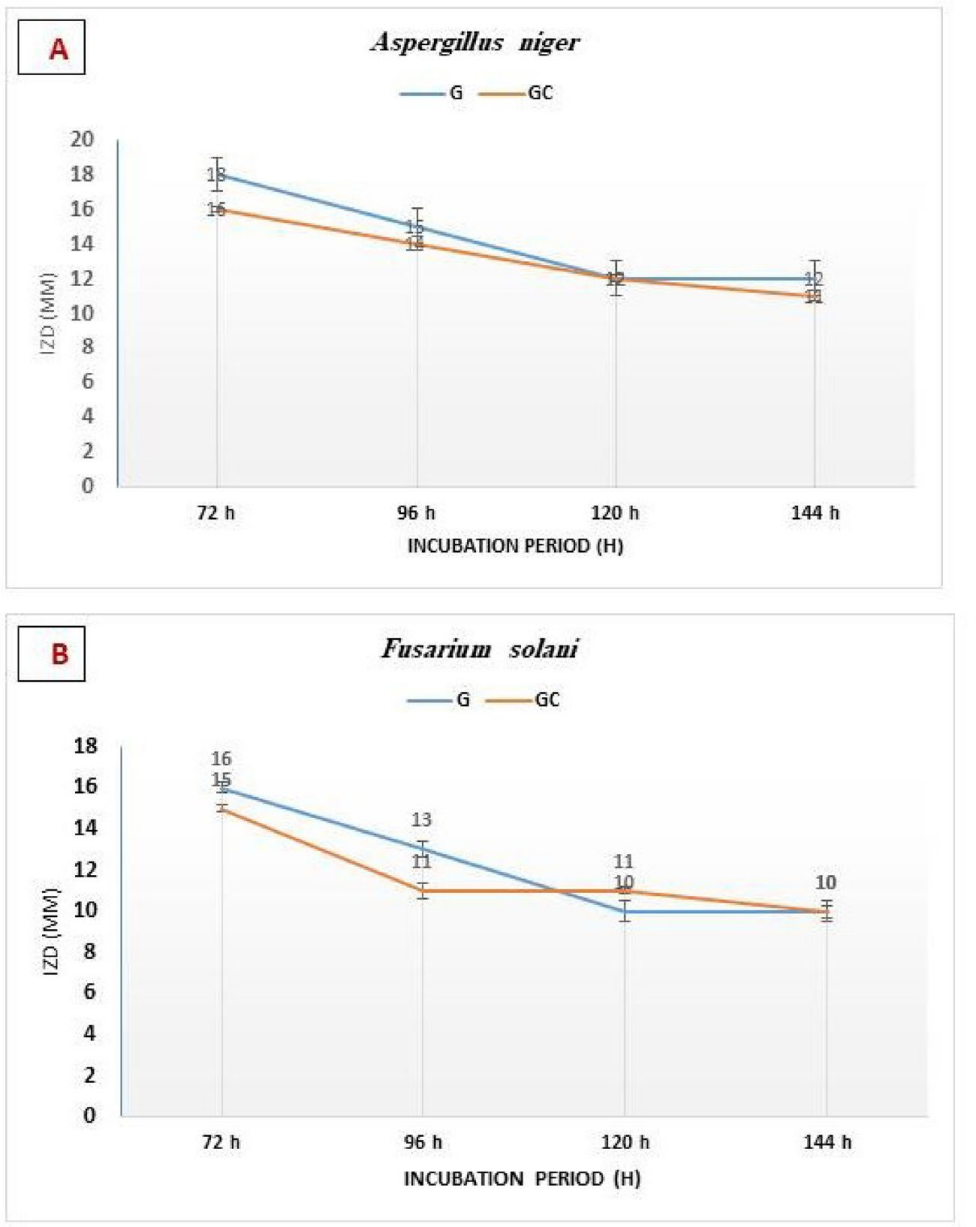
Antifungal stability test of W3Sr group of each glass and glass-ceramic samples against the pathogenic filamentous fungi *A. niger* (**A**) and *F. solani* (**B**). 100 mg/well (~ 9 mm diameter), with Sr²⁺ concentrations of 0.50 mg/mL. The data are shown as means ± SD of three independent experiments.

Antifungal stability was further confirmed by monitoring the inhibition zone diameters of the W3Sr formulation over a 6-days incubation period, during which the inhibition zones remained consistently present and quantifiable (Fig. 15). These findings are in agreement with the study of Maciel et al.^63^, who reported that partial substitution of Ca²⁺ with Sr²⁺ preserves antimicrobial activity, with even low Sr concentrations effectively inhibiting and eradicating microbial growth. Beyond its antimicrobial role, Sr incorporation has been shown to enhance the chemical durability and thermal stability of glass-based materials, although its direct antibacterial effect remains relatively modest^64^. Sr’s antimicrobial activity has been reported against a wide range of microorganisms, including oral pathogens^23^. Mechanistically, Sr²⁺ ions can penetrate microbial cells and disrupt critical cellular processes such as nucleic acid synthesis, protein production, and enzymatic activity. Additionally, Sr²⁺ interferes with ionic exchange pathways, leading to impaired cellular homeostasis and reduced microbial survival and proliferation^16^. These combined mechanisms direct biochemical disruption and ionic interference highlight the potential of Sr-containing materials for antifungal biomaterial application.

**Table 2:** Antimicrobial activity of glass samples.

Tested pathogens		Inhibition zone diameter (IZD- mm) at 100 mg/well sample			
		W0	W1Sr 0.125 mg/mL	W2Sr 0.25 mg/mL	W3Sr 0.50 mg/mL
Gram- positive bacteria	*Staphylococcus aureus* ATCC 6538	ND	ND	ND	ND
Gram-negative bacteria	*Escherichia coli* ATCC 25,922	ND	ND	ND	ND
Fungi	*Aspergillus niger* ATCC18666	ND	ND	12 ± 0.707	18 ± 0.707
	*Fusarium solani* NRC18	ND	ND	11 ± 0.707	16 ± 0.707

The data are shown as means ± SD of three independent experiments.

The agar diffusion method was employed, with the inhibition zone diameter (IZD) quantified in millimeters (mm).

ND.: Not detected.

**Table 3:** Antimicrobial activity of glass-ceramic samples.

Tested pathogens		Inhibition zone diameter (IZD-mm) at 100 mg/well sample			
		W0	W1Sr 0.125 mg/mL	W2Sr 0.25 mg/mL	W3Sr 0.50 mg/mL
Gram- Positive bacteria	*Staphylococcus aureus* ATCC 6538	ND	ND	ND	ND
Gram-negative bacteria	*Escherichia coli* ATCC 25,922	ND	ND	ND	ND
Fungi	*Aspergillus niger* ATCC18666	ND	ND	11 ± 0.707	16 ± 1.753
	*Fusarium solani* NRC18	ND	ND	10 ± 0.707	15 ± 1.703

The data are shown as means ± SD of three independent experiments.

The agar diffusion technique was followed and the inhibition zone diameter (IZD) expressed in (mm).

ND.: Not detected.

#### Cytotoxicity effect

**Fig. 16:**
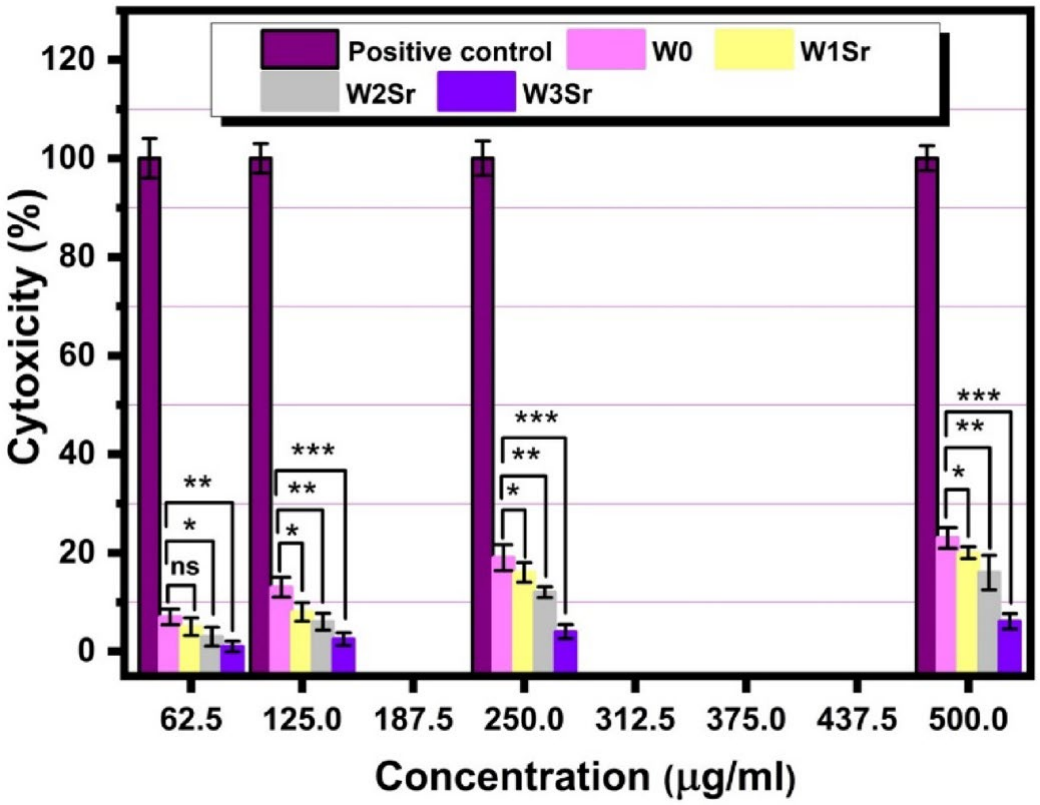
Cytotoxicity assessment of Sr-doped wollastonite ceramics (62.5–500 µg/mL) against human normal fibroblast (BJ1) cells after 48-hour exposure. Data are presented as mean ± SD (*n* = 3). Statistical significance relative to the undoped control (W0) is indicated: *p ≤ 0.05, **p ≤ 0.01, ***p ≤ 0.001; ns = not significant (*p* > 0.05).

Figure 16 demonstrates the cytotoxicity of Sr-doped wollastonite ceramics on human fibroblast (BJ1) cells after 48 h of exposure (62.5–500 µg/mL). All samples showed low cytotoxicity compared to the positive control (doxorubicin)^33^. The stark contrast in cytotoxicity results from their opposing functions: doxorubicin is a toxin designed to kill cells, while SrO-doped wollastonite is a bioactive ceramic designed to heal and support tissue growth. Notably, increasing Sr content enhanced biocompatibility, the cytotoxicity at 500 µg/mL dropping from 23% in pure wollastonite (W0) to 6.1% in the highest Sr-doped sample (W3Sr). This advocates that Sr incorporation enhances material safety for biomedical use. Strontium substitution mitigates the alkaline environment caused by excessive Ca²⁺ and Si⁴⁺ release from wollastonite, which can elevate pH and harm cells. Studies highlight that ions such as Ca²⁺ and Sr²⁺ are essential for promoting the adhesion of human bone-derived mesenchymal stem cells (MSCs)^65,66^. Instead, Sr²⁺ release at optimal concentrations (0.1–1 mM) promotes osteoblast activity, bone formation, and pH stabilization^15,66^. Based on our cytotoxicity results (Fig. 16), Sr-doped wollastonite ceramics demonstrate superior cytocompatibility compared with many ion-doped systems reported in the literature. Increasing the Sr content led to a clear reduction in cytotoxicity, with cell viability remaining high even at the maximum tested concentration (500 µg/mL). When compared with Ag-doped systems, which often show enhanced antimicrobial activity but are associated with increased cytotoxicity due to Ag⁺ ion release^59^ the Sr-doped wollastonite samples in this study maintain a safer and more cell-friendly response. Similarly, in contrast to Co-doped systems that may present concerns related to ion accumulation and long-term toxicity^51^, Sr-doped wollastonite offers a wider safety margin while still supporting cell growth. Additionally, *Palakurthy et al.*^42^, reveal that Sr-doped wollastonite ceramics outperform both pure wollastonite and control samples in promoting osteoblast proliferation. Strontium’s ability to enhance bone formation while minimizing resorption underscores its therapeutic potential. The study further confirms that Sr-enriched ceramics significantly boost osteoblast growth, aligning with evidence that Sr²⁺ ions strengthen bone integration and regeneration^8,67^.

**Fig. 17:**
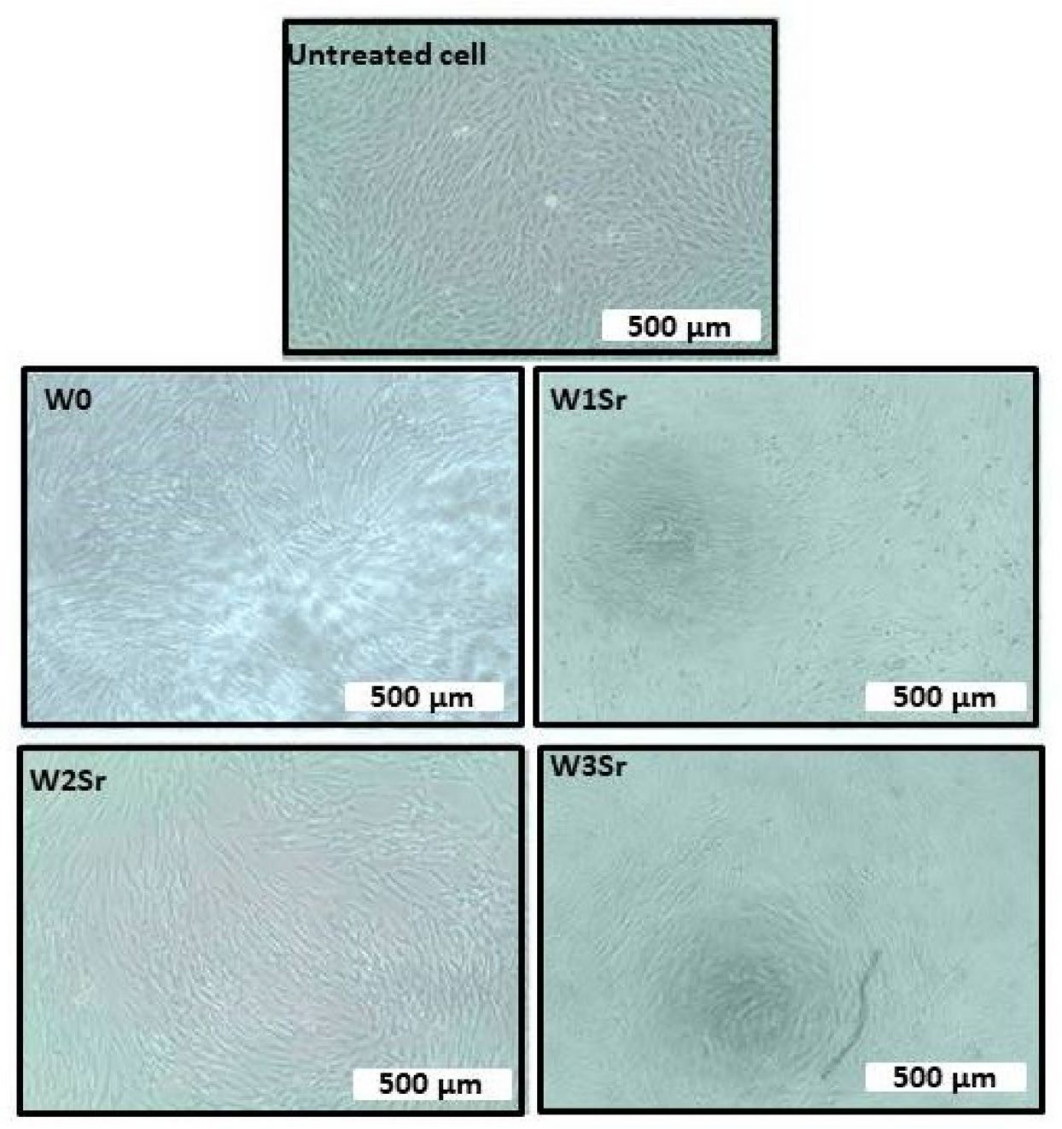
Morphological comparison of untreated cells and cells treated with 500 µg/mL at magnification 8000x.

As revealed in Fig. 17, no significant morphological changes were observed in BJ1 cells treated with 500 µg/mL of the material, confirming the biocompatibility of Sr-doped wollastonite. These results support its potential use in bone and dental applications, aligning with previous findings on the beneficial effects of Sr²⁺ on cell viability and tissue integration^8,23,67^.

## Conclusion

Strontium-doped wollastonite glass-ceramics, synthesized via melt-quenching, were systematically evaluated for their suitability in bone implant applications. Characterization revealed that Sr doping markedly promoted hydroxyapatite formation in simulated body fluid (SBF), as confirmed by XRD and FTIR analyses, which showed intensified phosphate peaks (P–O bending at 550 cm^-1^ and asymmetric stretching at 1036 cm⁻¹) with increasing Sr content, indicating enhanced HCA crystallization. This improved bioactivity achieved a bone-like Ca/P ratio of 1.70, while mechanical strength and bulk density increased significantly, reaching 77 MPa and 1.88 g/cm^2^, respectively, in the W3Sr formulation (0.5 wt% Sr), which demonstrated the best mechanical performance and biological efficacy. Although lacking antibacterial activity, the materials exhibited dose-dependent antifungal effects and excellent biocompatibility with BJI human fibroblasts. Overall, this study introduces a promising biomaterial that combines Sr-mediated bioactivity, improved mechanical properties, and selective antifungal activity, indicating considerable potential for advanced orthopedic and dental applications.

## Supplementary Information

Below is the link to the electronic supplementary material.

Supplementary Material 1

## Data Availability

The datasets generated and/or analyzed during the current study are not publicly available because they are private, but are available from the corresponding author on reasonable request.
