# Supplementary figures and images for: Characterization, in-vitro biological and antimicrobial testing of replacing Sr/Ca in wollastonite (Ca1 − x Srx SiO3) glass-ceramics
Source: Sci Rep. 2026 Feb 11;16:6347. doi: 10.1038/s41598-026-36649-1 (PMC12905248; doi:10.1038/s41598-026-36649-1)

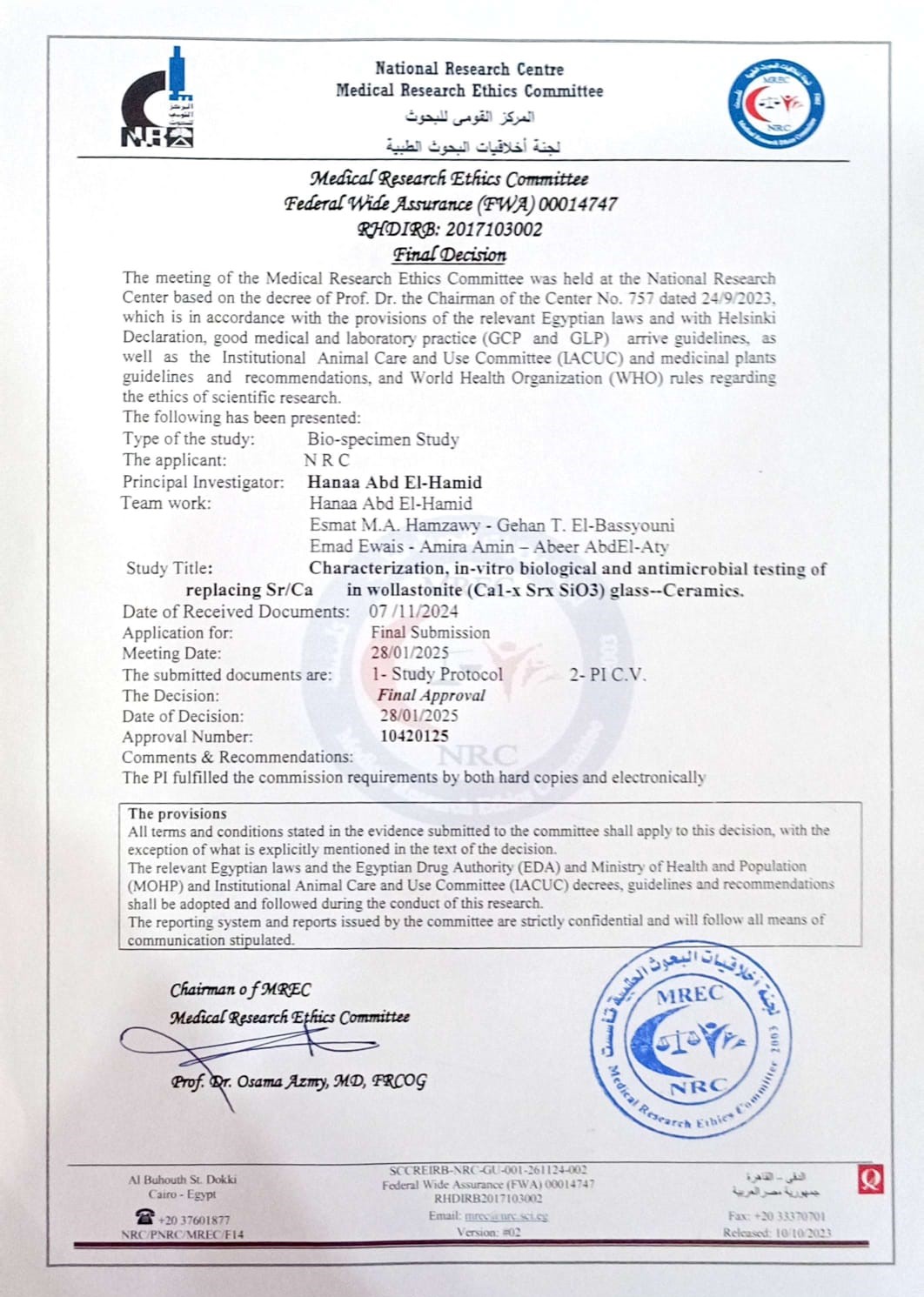

Supplement: Supplementary file 1 — Supplementary Material 1 [file 41598_2026_36649_MOESM1_ESM.jpeg]
